# Fat mass and obesity-associated protein regulates RNA methylation associated with depression-like behavior in mice

**DOI:** 10.1038/s41467-021-27044-7

**Published:** 2021-11-26

**Authors:** Shu Liu, Jianbo Xiu, Caiyun Zhu, Kexin Meng, Chen Li, Rongrong Han, Tingfu Du, Lanlan Li, Lingdan Xu, Renjie Liu, Wanwan Zhu, Yan Shen, Qi Xu

**Affiliations:** 1 State Key Laboratory of Medical Molecular Biology, Institute of Basic Medical Sciences Chinese Academy of Medical Sciences, School of Basic Medicine Peking Union Medical College, Beijing, 100005 China; 2 Neuroscience Center, Chinese Academy of Medical Sciences, Beijing, 100005 China

**Keywords:** Depression, RNA modification, Molecular neuroscience

## Abstract

Post-transcriptional modifications of RNA, such as RNA methylation, can epigenetically regulate behavior, for instance learning and memory. However, it is unclear whether RNA methylation plays a critical role in the pathophysiology of major depression disorder (MDD). Here, we report that expression of the fat mass and obesity associated gene (FTO), an RNA demethylase, is downregulated in the hippocampus of patients with MDD and mouse models of depression. Suppressing *Fto* expression in the mouse hippocampus results in depression-like behaviors in adult mice, whereas overexpression of FTO expression leads to rescue of the depression-like phenotype. Epitranscriptomic profiling of N6-methyladenosine (m^6^A) RNA methylation in the hippocampus of *Fto* knockdown (KD), *Fto* knockout (cKO), and FTO-overexpressing (OE) mice allows us to identify adrenoceptor beta 2 (*Adrb2*) mRNA as a target of FTO. ADRB2 stimulation rescues the depression-like behaviors in mice and spine loss induced by hippocampal *Fto* deficiency, possibly via the modulation of hippocampal SIRT1 expression by c-MYC. Our findings suggest that FTO is a regulator of a mechanism underlying depression-like behavior in mice.

## Abstract (web summary)

Post-transcriptional modification of RNA can contribute to regulating behavior. Here, the authors show that modulating the expression of *Fto* results in epitranscriptomic changes in the mouse hippocampus associated with depression-like behavior.

## Introduction

Major depressive disorder (MDD) is currently the leading cause of disability worldwide. MDD is characterized by low mood, diminished interests, feelings of despair or guilt, impaired cognition, and other symptoms^1^. Epigenetic regulation of the genes by interaction with environments contributes to the pathophysiology of depression^2^. Considerable progress has been made in the understanding of DNA methylation and histone modifications;^3^ however, the epitranscriptomic impact on depression remains largely elusive.

N6-methyladenosine (m^6^A) methylation that is the most abundant internal modification of eukaryotic mRNA occurs extensively in the brain^4,5^, and is dynamic and reversible in mammals^6^. FTO is a well-characterized RNA demethylase and is highly expressed in multiple brain regions^7^. Previous studies demonstrated that FTO in the brain could regulate postnatal growth of mice^8^, activity of the dopaminergic midbrain circuitry^9^, memory processes in the prefrontal cortex^10^ and the hippocampus^11–14^, brain development^12^, adult neurogenesis^12^, and axonal regeneration^15^. Several lines of evidence revealed that FTO was associated with depressive symptoms^16–18^ and involved in the pathogenesis of MDD^11,19^. Although these studies suggested that FTO could play a vital role in MDD, the mechanism behind specific induction of depression-like behaviors has yet to be clarified.

Here, we report that FTO in the hippocampus mediates depression-like behaviors through RNA demethylase activity. The expression of hippocampal FTO is decreased in patients with MDD and three mouse models of depression. Knockdown or knockout of *Fto* in the hippocampus induces depression-like behaviors, whereas overexpression of FTO has antidepressant effects. Epitranscriptomic analysis identifies ADRB2 likely to act as one of the targets modified by FTO. ADRB2 activation rescues depressive-like behaviors and spine loss caused by hippocampal FTO deficiency, and this effect is reversed by ADRB2 inhibition, suggesting the involvement of c-MYC in modulating hippocampal SIRT1 expression. It is conclusive that hippocampal FTO can be exploited to serve as a therapeutic target for depression.

## Results

### The expression of FTO is downregulated in the hippocampus of MDD patients and mouse models of depression

**Fig. 1:**
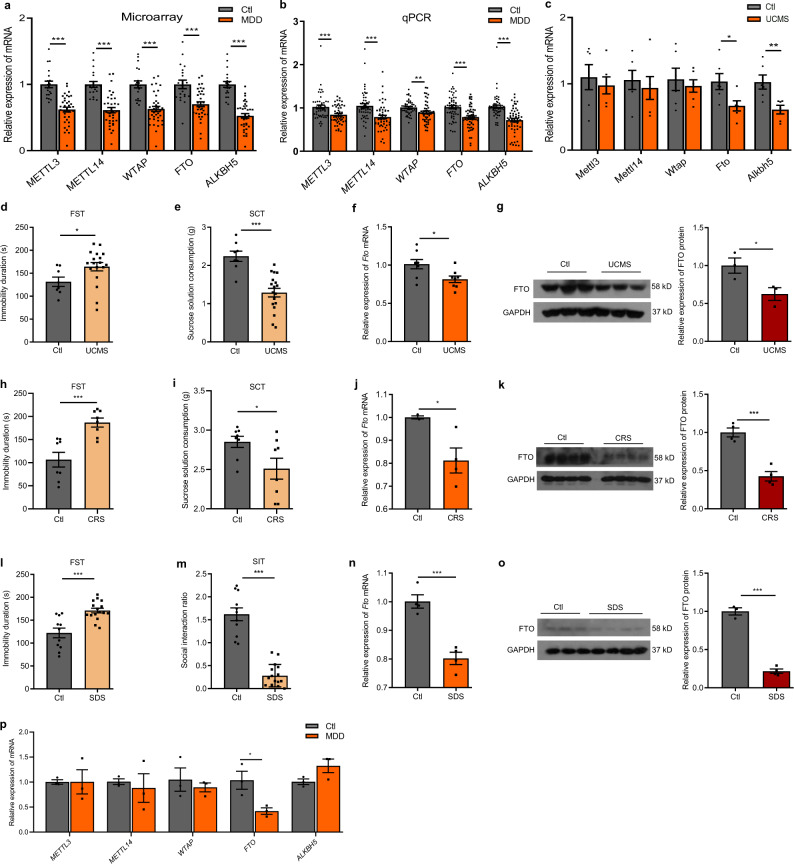
Expression of FTO is decreased in the hippocampus of MDD patients and mouse models of depression. **a** Microarray analysis of the genes encoding m^6^A-modifying enzymes in the peripheral blood of humans. Ctl, *n* = 20; major depressive disorder (MDD), *n* = 36. *METTL3*, *p* < 0.000001; *METTL14*, *p* < 0.000001; *WTAP*, *p* < 0.000001; *ALKBH5*, *p* < 0.000001; FTO, *p* = 0.000054. **b** Real-time quantitative PCR (qPCR) analysis of the genes encoding m^6^A-modifying enzymes in the peripheral blood of humans. Ctl, *n* = 50; MDD, *n* = 50. *METTL3*, *p* = 0.000755; *METTL14*, *p* = 0.000116; *WTAP*, *p* = 0.005480; *ALKBH5*, *p* < 0.000001; FTO, *p* = 0.000007. **c** mRNA expression of m^6^A-modifying enzymes in the peripheral blood of mice, *n* = 6 per group. UCMS, chronic unpredictable mild stress. *Mettl3*, *p* = 0.599250; *Mettl14*, *p* = 0.601975; *Wtap*, *p* = 0.614743; *Alkbh5*, *p* = 0.007390; FTO, *p* = 0.028276. **d**, **e** Forced swimming test (FST) and sucrose consumption test (SCT) in the UCMS mouse model. Ctl, *n* = 8; UCMS, *n* = 18. **d** *p* = 0.0432; **e** *p* < 0.0001. **f** Expression of hippocampal FTO mRNA in the UCMS model. *n* = 8 per group. *p* = 0.0182. **g** Expression of hippocampal FTO protein in the UCMS model. *n* = 3 per group. *p* = 0.0455. The samples derived from the same experiment and that blots were processed in parallel. **h, i** FST and SCT in the chronic restraint stress (CRS) mouse model. *n* = 8 per group. **h** *p* = 0.0007; **i** *p* = 0.03. **j** Expression of hippocampal FTO mRNA in the CRS model. Ctl, *n* = 3; CRS, *n* = 4. *p* = 0.037. **k** Expression of hippocampal FTO protein in the CRS model. *n* = 4 per group. *p* = 0.0005. **l**, **m** FST and social interaction test (SIT) in the social defeat stress (SDS) mouse model. Ctl, *n* = 11; SDS, *n* = 15. **l** *p* = 0.0004; **m** *p* < 0.0001. **n** Expression of hippocampal FTO mRNA in the SDS model. *n* = 4 per group. *p* = 0.0008. **o** Expression of hippocampal *Fto* protein in the SDS model. Ctl, *n* = 3; SDS, *n* = 4. *p* < 0.0001. **p** mRNA expression of m^6^A-modifying enzymes in the hippocampus of MDD patients. *n* = 3 per group. *METTL3*, *p* = 0.990981; *METTL14*, *p* = 0686706; *WTAP*, *p* = 0.565588; *ALKBH5*, *p* = 0.095860; FTO, *p* = 0.031700. *p < 0.05, **p < 0.01, ***p < 0.001. Two-tailed student’s t-test. Adjustments were made for multiple comparisons test. Error bars represent s.e.m. Source data are provided as a Source Data file.

In microarray analysis of the peripheral blood of 36 MDD patients and 20 healthy controls, we found that both RNA methyltransferases (*METTL3*, *METTL14*, and *WTAP*) and demethylases (FTO and *ALKBH5*) were significantly downregulated in the patient group (Fig. 1a). Next, we examined the expression of these genes in another cohort using real-time quantitative PCR and the results also showed that both RNA methyltransferases (*METTL3*, *METTL14*, and *WTAP*) and demethylases (FTO and *ALKBH5*) were significantly downregulated in MDD (Fig. 1b). We further measured the expression of m^6^A-modifying enzymes in three mouse models of depression, which have been widely employed in preclinical studies of depression, including the chronic unpredictable mild stress (UCMS), chronic restraint stress (CRS), and social defeat stress (SDS) models. The mRNA expression of *Fto* and *Alkbh5* in the peripheral blood of UCMS mice was significantly downregulated compared with control mice, whereas the expression levels of RNA methyltransferases remained unchanged (Fig. 1c). Behavioral tests showed that all three models of depression were successfully established (Fig. 1d, e, h, i, l, and m). We examined the mRNA expression of m^6^A-modifying enzymes in multiple brain regions involved in the regulation of emotions, including the prefrontal cortex (PFC), nucleus accumbens (NAC), amygdala (AMY), and hippocampus (HIP). We found that *Fto* was consistently downregulated in the hippocampus of all three animal models compared with the hippocampus of control mice (Fig. 1f, j, n), whereas there were either no changes or inconsistent changes in expression levels of the other enzymes examined (Supplementary Fig. 1a–c). Western blot analysis further confirmed that FTO was significantly downregulated in the hippocampus of all three mouse models compared with control mice (Fig. 1g, k, o). The postmortem study revealed that mRNA expression of FTO in the hippocampus of MDD patients was specifically and significantly decreased in the hippocampus of MDD patients compared with healthy controls (Fig. 1p, Supplementary Table 1).

### FTO mediates depression-like behaviors in the hippocampus

**Fig. 2:**
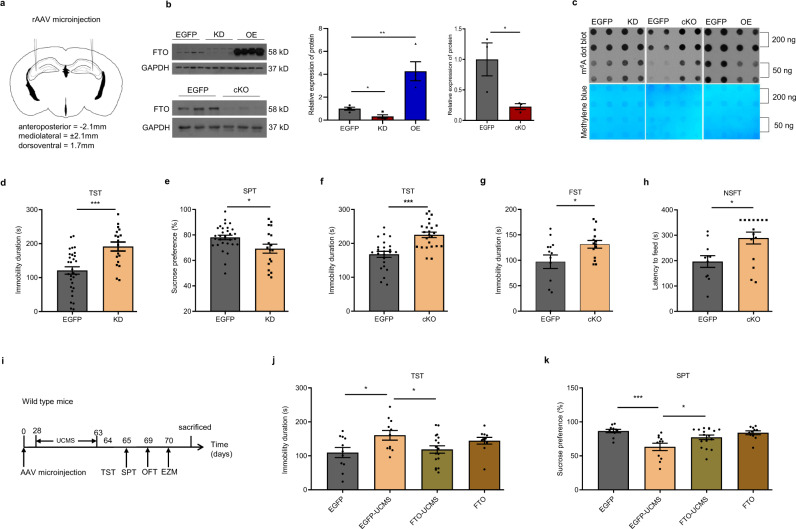
FTO in the hippocampus mediates depression-like behaviors. **a** Schematic illustrations of rAAV microinjection. **b** Knockdown (KD), overexpression (OE), and knockout (cKO) of *Fto* in the hippocampus. EGFP, KD, OE, *n* = 4 per group; EGFP, cKO, *n* = 3 per group. EGFP vs. KD, *p* = 0.0124; EGFP vs. OE, *p* = 0.0082; EGFP vs. cKO, *p* = 0.0476. **c** m^6^A dot blot assay of hippocampal total RNA. **d**, **e** Tail suspension test (TST) and sucrose preference test (SPT) in KD mice. Ctrl, *n* = 31; KD, *n* = 18. **d** *p* = 0.0002; **e** *p* = 0.0469. **f**–**h** TST, FST, and novelty suppressed feeding test (NSFT) in three groups of cKO mice. **f** *n* = 24 per group, *p* < 0.0001; **g** EGFP, *n* = 13; cKO, *n* = 14; *p* = 0.0324. **h** EGFP, *n* = 11; cKO, *n* = 15, *p* = 0.0193. **i** Schematics of the experimental design. **j**, **k** TST and SPT in OE mice subjected to UCMS. EGFP, EGFP-UCMS, FTO, *n* = 11 per group; FTO-UCMS, *n* = 17. **j** EGFP vs. EGFP-UCMS, *p* = 0.0193; EGFP-UCMS vs. FTO-UCMS, *p* = 0.0367; **k** EGFP vs. EGFP-UCMS, *p* = 0.0003; EGFP-UCMS vs. FTO-UCMS, *p* = 0.0162. *p < 0.05, **p < 0.01 ***p < 0.001. Two-tailed *t*-test for **b** and **d**–**h**; one-way ANOVA analysis of variance for **j** & **k**. Adjustments were made for multiple comparisons test. Error bars represent s.e.m. Source data are provided as a Source Data file.

To test how hippocampal FTO contributes to depression-like behaviors, we employed recombinant adeno-associated virus (rAAV) as a transgenic tool to specifically knock down mouse FTO overexpressed in the hippocampus of wild-type C57BL/6 mice and to specifically knock out *Fto* in the hippocampus of *Fto* flox/flox mice. Four weeks after bilateral microinjection of rAAV into the hippocampus (Fig. 2a), FTO protein showed an expected level in shRNA-expressing (knockdown; KD), FTO-overexpressing (OE), and Cre-expressing (cKO) mice (Fig. 2b). Given that FTO catalyzes RNA demethylation, the m^6^A dot blot assay showed that the alteration of FTO expression levels increased m^6^A levels in hippocampal total RNA from KD and cKO mice and decreased m^6^A levels in hippocampal total RNA from OE mice (Fig. 2c). Next, we analyzed the behavioral consequences of changes in *Fto* expression. KD mice demonstrated longer immobility duration in the tail suspension test (TST) and lower sucrose preference in the sucrose preference test (SPT) than control mice (Fig. 2d, e). Compared with EGFP-expressing mice, cKO mice showed longer duration of immobility in the TST and the forced swimming test (FST) as well as longer latency to feeding in the novelty suppressed feeding test (NSFT) (Fig. 2f, g, h). These results suggest that suppressing *Fto* expression in the hippocampus is sufficient to induce depression-like behaviors in mice. Subsequently, we explored whether overexpression of FTO could lead to antidepressant effects. UCMS induced depression-like behaviors in EGFP-expressing mice, but stress-treated OE mice demonstrated shorter immobility duration in the TST and higher sucrose preference in the SPT than UCMS-treated EGFP-expressing mice (Fig. 2i–k). These results suggest that overexpression of FTO in the hippocampus has antidepressant effects.

Recent studies have reported various effects of systemic *Fto* knockout on anxiety-like behaviors in mice^13,19^. Owing to the high comorbidity of depression and anxiety, we also analyzed the anxiety-like behaviors in KD and cKO mice. There was no significant difference in the time spent in the center zone in the open field test (OFT) between KD and control mice (Supplementary Fig. 2a). Additionally, in the elevated zero maze test (EZM), there was no significant difference in the time spent in the open arms between KD and control mice (Supplementary Fig. 2b). Similar results were observed in cKO mice (Supplementary Fig. 2c, d). These results are consistent with those from a previous study that used a specific Cre line to knock out *Fto* in the CA1 and CA3 regions of the hippocampus^11^. Our study demonstrated that FTO in the hippocampus specifically mediated depression-like behaviors without affecting anxiety-like behaviors. Because specific deletion of *Fto* in the nervous system has been found to result in postnatal growth retardation^8^, we examined whether the depression-like phenotype observed in cKO mice was developed due to abnormal growth. However, there was no significant difference in body weight between control and cKO mice, indicating that downregulation of *Fto* in the hippocampus did not affect growth (Supplementary Fig. 3a). Moreover, after 24 h of food deprivation, there was no change in body weight between the two groups of mice (Supplementary Fig. 3b, c).

### Modification of the hippocampal m^6^A epitranscriptome by FTO

**Fig. 3:**
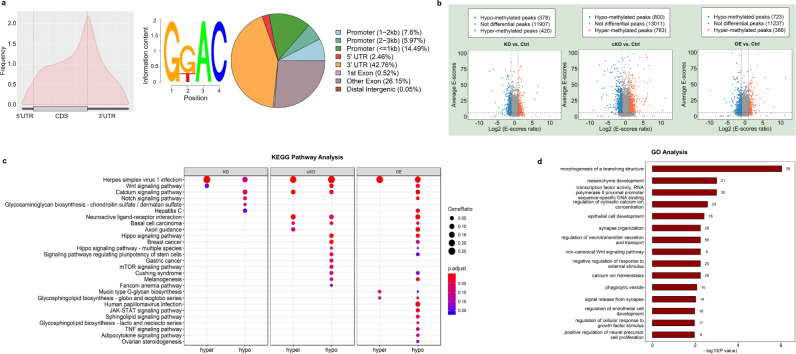
Hippocampal m^6^A epitranscriptome modified by FTO. **a** Left: distribution of m^6^A peaks throughout the whole mRNA transcript; middle: the most abundant motifs detected in peaks and enriched at peak summits; right: annotation of peaks detected in cKO mice. **b** Differentially methylated peaks among different groups. **c** KEGG pathway analysis of differentially methylated genes in different groups. **d** GO analysis of genes with hypomethylated peaks in OE mice.

Next, we focused on investigating the mechanism by which hippocampal FTO mediates depression-like behaviors. We performed m^6^A epitranscriptomic analysis of hippocampal mRNAs by MeRIP-Seq. After quality control, 47.01 to 76.53 million reads were generated from each m^6^A-seq library, 88.59% of which were uniquely mapped to the mouse genome and retained for further analysis (Supplementary Table 2). Approximately 25%–34% of the expressed genes were modified by m^6^A, with approximately 2.65 peaks per gene. The pattern of m^6^A modification distribution across the genes was similar among all groups (Supplementary Fig. 4a). Consistent with previous studies^5,20^, the majority of m^6^A peaks were preferentially located in the 3’UTR and around the stop codon, and the enriched motifs in all samples contained ‘GGAC’, one of the most frequent m^6^A consensus motifs (Fig. 3a).

Next, we analyzed the differentially methylated peaks (DMPs) among different groups (Fig. 3b). Most DMPs were distributed in exons and the 3’UTR (Supplementary Fig. 4b). KEGG pathway analysis showed that multiple gene clusters were enriched in Wnt, calcium, Notch, and axon guidance signaling pathways (Fig. 3c). Specifically, hypermethylated genes in cKO mice and hypomethylated genes in OE mice were both enriched in the neuroactive ligand-receptor interaction pathway. GO analysis demonstrated that the genes with hypomethylated peaks in OE mice were mostly enriched in the pathways regulating synapse organization and signal release, neurotransmitter secretion and transport, and neural precursor cell proliferation (Fig. 3d).

### ADRB2 is a target gene of FTO in the hippocampus

**Fig. 4:**
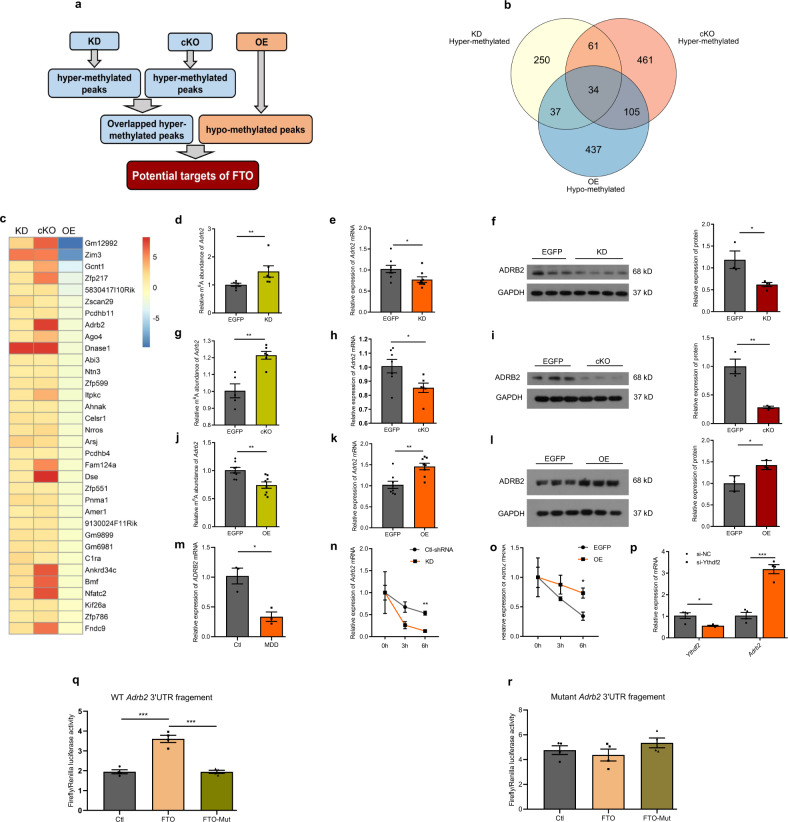
ADRB2 is one of the critical target genes of FTO in the hippocampus. **a** Schematics of the strategy used to identify the targets. **b** Overlap of the genes that are hypermethylated in KD and cKO mice and hypomethylated in OE mice. **c** Thirty-four genes that are hypermethylated in KD and cKO mice and hypomethylated in OE mice. **d**–**l** m^6^A, mRNA, and protein levels of *Adrb2* in the hippocampus of KD (**d**–**f**) cKO (**g**–**i**) and OE (**j**–**l**) mice. **d** *n* = 6 per group, *p* = 0.0087; **e** *n* = 8 per group, *p* = 0.0381; **f** EGFP, *n* = 3; KD, *n* = 4, *p* = 0.0243; **g** EGFP, *n* = 5; cKO, *n* = 6, *p* = 0.0012; **h** EGFP, *n* = 7; cKO, *n* = 6, *p* = 0.0269; **i** *n* = 3 per group, *p* = 0.0053; **j** *n* = 8 per group, *p* = 0.0032 **k** *n* = 8 per group, *p* = 0.0022; **l** *n* = 3 per group, *p* = 0.0232. The detection of ADRB2 in panel l was in the same experiment as the detection of SIRT1 in OE in panel e of Fig. 5. **m** Expression of *Adrb2* mRNA in the hippocampus of MDD patients and healthy controls. *n* = 3 per group, *p* = 0.0115. **n**, **o** *Adrb2* mRNA stability assay after transfection of Neuro-2a cells with plasmids expressing *Fto*-shRNA or *Fto*. *n* = 4 per time point. **n** Ctl-shRNA vs. KD, 6 h, *p* = 0.0053; **o** EGFP vs. OE, 6 h, *p* = 0.0383. **p** Levels of *Ythdf2* and *Adrb2* mRNA after knockdown of *Ythdf2* in Neuro-2a cells. *n* = 4 per group. *Ythdf2*, *p* = 0.0130003; *Adrb2*, *p* = 0.000153. **q**, **r** Relative luciferase activity of pMIR-GLO-*Adrb2*-3′UTR, with either wild-type (WT *Adrb2* 3' UTR fragement) or mutant m^6^A sites (Mutant Adrb2 3' UTR fragement) after co-transfection with control vector (Ctl), FTO (FTO), or FTO-mutant (FTO-Mut) into HEK293T cells for 48 h. Firefly luciferase activity was measured and normalized to Renilla luciferase activity, *n* = 4 per group. **q** Ctl vs. FTO, *p* = 0.0002; FTO vs. FTO-Mut, *p* = 0.0002 **r** Ctl vs. FTO, *p* = 0.5376; FTO vs. FTO-Mut, *p* = 0.1666. *p < 0.05, **p < 0.01, ***p < 0.001. Two-tailed Student’s *t*-test for **d**–**m** & **p**; two-way ANOVA with repeated measures followed by post hoc Tukey’s test for **n** & **o**. Adjustments were made for multiple comparisons test. Error bars represent s.e.m. Source data are provided as a Source Data file.

Given that FTO is a well-characterized RNA demethylase, we analyzed hypermethylated mRNAs in KD and cKO mice, and hypomethylated mRNAs in OE mice (Fig. 4a). After mapping each peak to the corresponding gene, we found that 382 and 661 genes were hypermethylated in KD and cKO mice, respectively, while there were 613 hypomethylated genes in OE mice (Fig. 4b). Finally, we identified 34 genes that were both hypermethylated in KD and cKO mice and hypomethylated in OE mice (Fig. 4c; Supplementary Table 3). Gene-specific m^6^A qPCR analysis of *Zfp217* and *Ahnak* confirmed the MeRIP-Seq results (Supplementary Fig. 4c). At the mRNA level, *Zfp217* and *Ahnak* were downregulated in cKO mice and upregulated in OE mice compared with control mice (Supplementary Fig. 4d). Among these 34 genes, *Adrb2* encodes the β2-adrenergic receptor, which has been reported to regulate synaptic plasticity, adult neurogenesis and neuroinflammation in the hippocampus, and exerts antidepressant effects^21–25^. Therefore, we further examined whether ADRB2 contributed to FTO-mediated depression-like behaviors in the hippocampus. First, we confirmed that suppressing *Fto* expression hyper-methylated *Adrb2* mRNA in KD and cKO mice, while FTO overexpression led to hypomethylation of *Adrb2* mRNA in OE mice (Fig. 4d, g, j). At both the mRNA and protein levels, *Adrb2* was downregulated in KD and cKO mice but upregulated in OE mice compared to control mice (Fig. 4e, f, h, i, k, and l). The postmortem study revealed that mRNA expression of *Adrb2* was significantly lower in the hippocampus of MDD patients than healthy controls (Fig. 4m). Next, we performed luciferase reporter assays to determine whether ADRB2 was the direct target of FTO. Wild-type FTO increased luciferase activity of pMIR-GLO vectors bearing *Adrb2*−3′UTR with wild-type m^6^A sites compared with empty vector or mutant FTO vector. In contrast, wild-type FTO did not influence the activity of pMIR-GLO vectors bearing *Adrb2* 3′UTR with mutant m^6^A sites (Fig. 4q, r). We further investigated whether the changes in m^6^A methylation affected *Adrb2* mRNA levels in Neuro-2a cells. In the presence of actinomycin D, an inhibitor of mRNA transcription, *Fto* knockdown accelerated the degradation of *Adrb2* mRNA, whereas FTO overexpression significantly delayed this process (Fig. 4n, o). When YTHDF2, an m^6^A reader protein that regulates RNA stability^26^, was knocked down, the *Adrb2* mRNA levels were significantly increased (Fig. 4p). These results indicate that m^6^A modification is likely to affect the *Adrb2* mRNA levels, at least partly by regulating its stability.

### ADRB2 activation reverses the depression-like behaviors and spine loss induced by hippocampal *Fto* deficiency

**Fig. 5:**
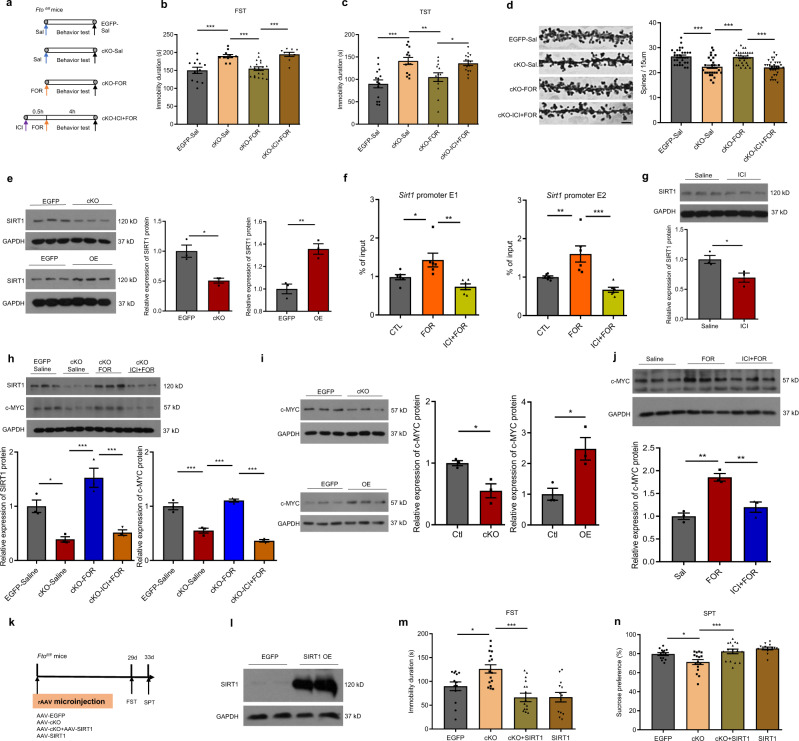
ADRB2 stimulation rescues depression-like behaviors and spine loss induced by hippocampal FTO deficiency. **a** Schematics of the experimental design. Sal, Saline; FOR, formoterol, a specific Adrb2 agonist; ICI, ICI 118,551, a specific Adrb2 antagonist. **b** FST in one group of cKO mice. EGFP-Sal, *n* = 12; cKO-Sal, *n* = 11; cKO-FOR, *n* = 19; cKO-ICI + FOR, *n* = 9. EGFP-Sal vs. cKO-Sal, *p* = 0.0003; cKO-Sal vs. cKO-FOR, *p* = 0.0004, cKO-FOR vs. cKO-ICI + FOR, *p* = 0.0002. **c** TST in another group of cKO mice. EGFP-Sal, *n* = 15; cKO-Sal, *n* = 13; cKO-FOR, *n* = 13; cKO-ICI + FOR, *n* = 15. EGFP-Sal vs. cKO-Sal, *p* < 0.0001; cKO-Sal vs. cKO-FOR, *p* = 0.0090, cKO-FOR vs. cKO-ICI + FOR, *p* = 0.0245. **d** Spine density analysis in the hippocampus of cKO mice after seven days of ADRB2 activation. *n* = 30 per group. Scale bar = 2 μm. EGFP-Sal vs. cKO-Sal, *p* < 0.0001; cKO-Sal vs. cKO-FOR, *p* < 0.0001, cKO-FOR vs. cKO-ICI + FOR, *p* < 0.0001. **e** SIRT1 expression in the hippocampus of cKO and OE mice. *n* = 3 per group. EGFP vs. cKO, *p* = 0.0110; EGFP vs. OE, *p* = 0.0049. **f** ChIP-qPCR assay performed on N2A cells treated with FOR or ICI + FOR. Expression of promoter of Sirt1 with primers from −1009 to −850 bp (E1) or from −2535 to −2385 bp (E2). *n* = 6 per group. E1, CTL vs. FOR, p = 0.035; FOR vs. ICI + FOR, *p* = 0.0017; E2, CTL vs. FOR, *p* = 0.0094; FOR vs. ICI + FOR, *p* = 0.0002. **g** SIRT1 expression in the hippocampus of wild-type mice treated with ICI or saline for 4.5 h. *n* = 3 per group, *p* = 0.0406. The samples derived from the same experiment and that blots were processed in parallel. **h** SIRT1 and c-MYC expression in the hippocampus of cKO and control mice after ADRB2 activation. *n* = 3 per group. SIRT1, EGFP-Saline vs. cKO-Saline, *p* = 0.0143; cKO-Saline vs. cKO-FOR, *p* = 0.0003; cKO-FOR vs. cKO-ICI + FOR, *p* = 0.0006. c-MYC, EGFP-Saline vs. cKO-Saline, *p* = 0.0002; cKO-Saline vs. cKO-FOR, *p* < 0.0001; cKO-FOR vs. cKO-ICI + FOR, *p* < 0.0001. **i** c-MYC expression in the hippocampus of cKO and OE mice. *n* = 3 per group. Ctl vs. cKO, *p* = 0.0203; Ctl vs. OE, *p* = 0.0236. **j** c-MYC expression in the hippocampus of wild-type mice after ADRB2 activation. *n* = 3 per group. Sal vs. FOR, *p* = 0.0011; FOR vs. ICI + FOR, *p* = 0.0045. **k** Schematics of the experimental design. **l** SIRT1 overexpression in the hippocampus. *n* = 2 per group. **m**, **n** FST and SPT after elevation of Sirt1 expression in the hippocampus of cKO and control mice. EGFP, *n* = 13; cKO, *n* = 16; cKO+SIRT1, *n* = 16; SIRT1, *n* = 13. **m** EGFP vs. cKO, *p* = 0.0137; cKO vs. cKO+SIRT1, *p* < 0.0001. **n** EGFP vs. cKO, *p* = 0.0166; cKO vs. cKO+SIRT1, *p* = 0.0006. *p < 0.05, **p < 0.01, ***p < 0.001. Two-tailed *t*-test for **e**, **g** and **i**; one-way ANOVA for **b**–**d**, **f**, **h**, **j**, **m** and **n**. Adjustments were made for multiple comparisons test. Error bars represent s.e.m. Source data are provided as a Source Data file.

We investigated whether ADRB2 affected the phenotype induced by hippocampal *Fto* deficiency. We activated ADRB2 in cKO mice with formoterol (FOR), a specific ADRB2 agonist that can cross the blood-brain barrier (Fig. 5a). FOR administration rescued the depression-like behaviors of cKO mice in both the FST and TST in two groups of mice, while pretreatment with ICI 118,551 (ICI), a specific ADRB2 antagonist, blocked the effects of FOR (Fig. 5b, c). Specifically, hippocampal administration of FOR reversed the depression-like phenotype of *Fto* cKO mice (Supplementary Fig. 5a–c). Depression has been demonstrated to be linked to synaptic dysfunction in multiple brain regions, including the hippocampus^27^. The cKO mice treated with FOR for seven consecutive days significantly increased the number of spines to a level comparable to that in EGFP-expressing mice, whereas pretreatment with ICI blocked these effects of FOR (Fig. 5d).

### ADRB2 mediates depression-like behaviors possibly via the modulation of hippocampal SIRT1 expression by c-MYC

**Fig. 6:**
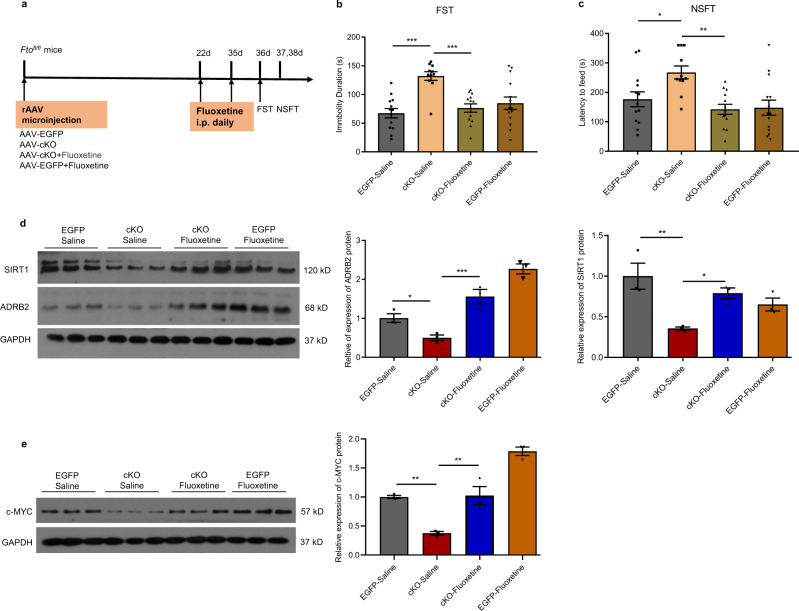
Fluoxetine rescues depression-like behaviors and ADRB2/c-MYC/SIRT1 protein expression induced by hippocampal FT*O* deficiency. **a** Schematics of the experimental design. **b**, **c** FST and NSFT after treatment with fluoxetine. EGFP-Saline, *n* = 13; cKO-Saline, *n* = 11; cKO+Fluoxetine, *n* = 13; EGFP- Fluoxetine, *n* = 14. **b** EGFP-Saline vs. cKO-Saline, *p* < 0.0001; cKO-Saline vs. Cko+Fluoxetine, *p* = 0.0001. **c** EGFP-Saline vs. cKO-Saline, *p* = 0.0187; cKO-Saline vs. Cko+Fluoxetine, *p* = 0.0011. **d**, **e** SIRT1, ADRB2 and c-MYC expression in the hippocampus of *Fto*-cKO mice treated with fluoxetine, *n* = 3 per group. **d** ADRB2, EGFP-Saline vs. cKO-Saline, *p* = 0.0481; cKO-Saline vs. cKO+Fluoxetine, *p* = 0.0008. SIRT1, EGFP-Saline vs. cKO-Saline, *p* = 0.0028; cKO-Saline vs. cKO+Fluoxetine, *p* = 0.0239. **e** EGFP-Saline vs. cKO-Saline, *p* = 0.0021; cKO-Saline vs. cKO+Fluoxetine, *p* = 0.0016. *p < 0.05, **p < 0.01, ***p < 0.001. Statistical significance was calculated using one-way ANOVA and adjustments were made for multiple comparisons test. Source data are provided as a Source Data file.

SIRT1, an NAD^+^-dependent deacetylase, is essential for synaptic plasticity in the hippocampus^28,29^, and it has been reported that hippocampal SIRT1 signaling mediates depression-like behaviors^30^. In this study, we found that the SIRT1 protein was downregulated in the hippocampus of cKO mice but upregulated in OE mice (Fig. 5e). However, in all three m^6^A-seq libraries, we detected no changes in the m^6^A level on *Sirt1* mRNA, suggesting that FTO may regulate the expression of SIRT1 through other pathways. Recent studies have reported that ADRB2 activation upregulates SIRT1 expression in cervical cancer cells through promoting the expression of c-MYC^31^. In vitro, c-MYC can bind to the *Sirt1* promoter and induce *Sirt1* expression^32^. Our CHIP assay confirmed that c-MYC were bound to the promoter of Sirt1^33^. Stimulation of ADRB2 significantly increased the abundance of c-MYC at the binding site of the *Sirt1* promoter. Pretreatment with ICI blocked the effects of FOR on the abundance of c-MYC at the binding site of the *Sirt1* promoter (Fig. 5f). Blocking ADRB2 significantly decreased SIRT1 protein levels in the hippocampus of wild-type mice after intraperitoneal administration of ICI (Fig. 5g). SIRT1 expression levels were obviously increased in FOR-treated cKO mice compared with saline-treated cKO mice, while pretreatment with ICI blocked the effects of FOR (Fig. 5h). Similar finding of SIRT1 expression was observed in c-MYC in OE or cKO mice (Fig. 5i). The results from specific administration of FOR into the hippocampus were also similar to those from intraperitoneal administration. FOR treatment obviously increased hippocampal SIRT1 and c-MYC expression in cKO mice compared with saline-treated cKO mice (Supplementary Fig. 5d, e). There was also no change in the m^6^A level on *c-Myc* mRNA in the KD, cKO, and OE m^6^A-seq libraries. FOR treatment also increased c-MYC expression in cKO mice compared with saline-treated cKO mice, while pretreatment with ICI blocked the effects of FOR (Fig. 5h). FOR-activated ADRB2 significantly increased c-MYC protein expression in wild-type mice compared with saline-treated mice, and this effect was blocked by pretreatment with ICI (Fig. 5j). Finally, overexpression of SIRT1 in the hippocampus of cKO mice effectively reversed the depression-like behaviors induced by *Fto* deficiency (Fig. 5k–n). These observations suggest that ADRB2 regulates downstream of FTO in the hippocampus to mediate depression-like behaviors and that ADRB2 may target SIRT1 through modulating the expression of c-MYC. Further, we investigated whether fluoxetine, the most widely prescribed antidepressant in clinical practice, could improve the depression-like behaviors in *Fto* cKO mice and whether the ADRB2-c-MYC-SIRT1 signaling pathway was involved in the actions of fluoxetine. We found that fluoxetine treatment significantly reversed the depression-like phenotype of *Fto* cKO mice (Fig. 6a–c). The expression levels of ADRB2, SIRT1, and c-MYC in the hippocampus were significantly higher in fluoxetine-treated than saline-treated cKO mice (Fig. 6d, e).

## Discussion

**Fig. 7:**
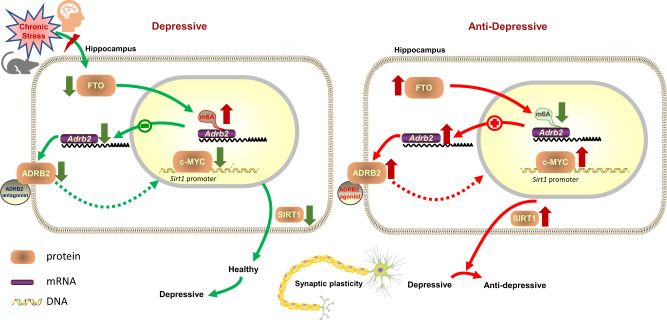
Schematic illustration summarizing the current work. FTO expression is downregulated in the hippocampus of both patients with major depressive disorder (MDD) and chronic stress-induced mouse models of depression. Downregulation of FTO leads to elevated m^6^A modification level of *Adrb2* mRNA, which results in lower mRNA and protein expression of *Adrb2*; in contrast, enhancing FTO expression in the hippocampus reduces the m^6^A modification level of *Adrb2* mRNA, which results in higher expression levels of *Adrb2* mRNA and protein. The activation of ADRB2 can increase the expression of c-MYC, while pretreatment with an ADRB2 antagonist blocks the effects. In vitro, c-MYC can bind to the *Sirt1* promoter. The activation of ADRB2 also increases the expression of SIRT1, while pretreatment with the antagonist blocks the effects. However, how the signal is transduced from ADRB2 to c-MYC has yet to be clarified. ADRB2 activation reverses the depression-like behaviors and spine loss induced by FTO deficiency in the hippocampus. Overexpression of SIRT1 in the hippocampus also reverses the depression-like behaviors in *Fto* cKO mice. The picture material was downloaded from ScienceSlides (http://www.scienceslides.com).

According to our findings, the expression of hippocampal FTO was decreased in both patients with MDD and the three mouse models of depression. Specific knockdown or knockout of *Fto* in the hippocampus could induce depression-like behaviors, whereas overexpression of FTO had antidepressant effects. Epitranscriptomic analysis identified ADRB2 as one of the targets modified by FTO. Loss of *Fto* in the hippocampus decreased the expression of ADRB2 by elevating m^6^A level in *Adrb2* mRNA. Conversely, overexpression of FTO could increase the expression of ADRB2 by reducing m^6^A level in *Adrb2* mRNA. ADRB2 stimulation could rescue depressive-like behaviors and spine loss caused by hippocampal FTO deficiency, which can be reversed by ADRB2 inhibition and may function via c-MYC to modulate hippocampal SIRT1 expression (Fig. 7). Interestingly, downregulation of hippocampal FTO did not affect anxiety-like behaviors that are often observed in stress-induced models of depression, including those employed in the current study. Although anxiety disorders and MDD are highly co-morbid, they are traditionally defined as two distinct diseases^34^. At present, nonanxious MDD is distinguished from anxious MDD based on the cutoff score <7 for anxiety/somatization factor of Hamilton Depression Rating Scale (HAM-D) 17^35,36^. Particularly, anxious MDD has a poorer response to antidepressants than nonanxious MDD, consistent with our antidepressant treatment of *Fto* knockout in hippocampus. While most of the existing depression models are comorbid with anxiety, our model of FTO deficiency in the hippocampus may serve as a model of nonanxious MDD. It would be interesting to further investigate the differential mechanisms of anxious versus nonanxious depression by comparing common depression models and the hippocampal FTO deficiency model. Genetic studies have shown that the FTO rs9939609 variant exerts a protective effect against depression;^17^ further analyses have revealed that this positive association is driven by an atypical MDD subtype^16^. However, the role of FTO in the pathophysiology of depression remains largely unknown. Inconsistent results regarding anxiety phenotypes found in different *Fto* mutant mice^11,13,19^ highlight the regional specificity of the functions of FTO in the brain, such as modulation of dopaminergic signaling in the midbrain and regulation of fear memory in the prefrontal cortex. The present study demonstrated that FTO in the hippocampus was critical in mediating depression-like behaviors.

MeRIP-seq assay and functional study confirmed that ADRB2 was an important target gene of FTO. Although FTO may target many mRNAs, our study focused on *Adrb2* mRNA because ADRB2 can regulate synaptic plasticity and contribute to many mental disorders^22–24,37^. Previous studies in animals have reported that stimulation of central beta 2 -adrenergic receptors can produce antidepressant-like effects^38^. While a large number of studies indicate that activation of central ADRB2 can produce antidepressant-like effects on animals, the specific mechanisms remain unclear. In this study, however, we found that depleted expression of FTO increased m6A levels at 3′UTRs, leading to the downregulation of ADRB2 at both the RNA and protein levels. In contrast, the overexpression of FTO in the hippocampal tissues decreased m6A level and increased the expression of ADRB2. Wang et. al reported that m6A-containing mRNA was less stable because such mRNA could be relocalized to the decay sites by YTHDF2^26^. According to our results, *Fto* knockdown accelerates the degradation of *Adrb2* mRNA, and overexpression of FTO alleviates the degradation of *Adrb2* mRNA. Taken together, the regulation of ADRB2 expression by FTO relies on demethylase activity to a certain extent.

Two loci, one of which is near the *SIRT1* gene, were identified as the first robust links between genetics and depression at the whole-genome level^39^. Recent studies reported that the expression of *SIRT1* in the peripheral blood of MDD patients was significantly lower in patients with MDD than healthy controls^40^, consistent with our microarray analysis (in-house data). Animal studies have also shown that SIRT1 regulates depression-like behaviors in several brain regions, including the hippocampus^30,41–43^. We observed that the methylation level of *Sirt1* mRNA was not modified by FTO but the expression level of SIRT1 protein was altered by FTO. Chen et al found that ADRB2 activation upregulated the expression of Sirt1 via elevating c-MYC expression in cervical cancer cells^31^. c-MYC is a transcriptional factor of *Sirt1* and can regulate the expression of SIRT1 in vitro^44^. Consistent with previous results, we found that injection of FOR increased the expression of c-MYC protein in the hippocampus whereas injection of ICI blocked this effect. In addition, the expression of c-MYC protein was decreased in the hippocampus of *Fto* cKO mice and increased in the hippocampus of FTO overexpression mice. We report that ADRB2 regulates SIRT1 expression in vivo in the hippocampus of wild-type and *Fto*-deficient mice. Both the activation of ADRB2 and the elevation of hippocampal SIRT1 levels reversed the depression-like behaviors induced by *Fto* deficiency, suggesting that FTO may target SIRT1 through modifying the methylation of *Adrb2* mRNA. The limitation of the study is that only male mice were employed. In light of the established fact that mood disorders (such as MDD) have a higher incidence in women, therefore further work investigating the role of FTO in female mice may help elucidate potential sex-specific mechanisms underlying mood disorders.

Our study indicates that FTO in the hippocampus mediates depression-like behaviors and that hippocampal FTO can be exploited to serve as a therapeutic target for depression. Recently developed specific FTO inhibitors are promising for the treatment of cancer^45^ and obesity^46^. Based on our findings from several weight-loss drugs^47^, an attention should be paid to potential adverse psychiatric events during systematic administration of FTO inhibitors.

## Methods

All the subjects provided written informed consent to participate in this study that was approved by the Ethics Committee of the Chinese Academy of Medical Sciences and Peking Union Medical College. Hippocampal tissues of the human brain were provided by the Human Brain Bank, Chinese Academy of Medical Sciences & Peking Union Medical College, Beijing, China. The animal experiments were in accordance with the institutional guidelines of the Beijing Administration Office of Laboratory and this study was approved by the Institutional Review Board of Chinese Academy of Medical Sciences and Peking Union Medical College. The protocol number is 2019017. The donation of human tissue was voluntary and free, approved by the donor and all the donor’s direct family members. It was also approved that the donated human tissue will only be used for scientific research.

### Human subjects

Thirty-six patients with MDD (20 women and 16 man) aged 39.9 ± 12.5 (SD) years and 20 healthy controls (10 women and 10 man) aged 36.6 ± 11.1 (SD) years were recruited through the Department of Psychiatry, First Hospital of Shanxi Medical University, Taiyuan, China. Fifty patients with MDD (27 women and 23 men) aged 31.5 ± 9.8 (SD) years and 50 healthy controls (28 women and 22 men) aged 29.5 ± 8.3 (SD) years were recruited to validate the result of microarray. None of these patients with MDD or healthy controls had taken any psychotropic medications within 4 weeks. All of the patients were clinically diagnosed by at least two psychiatrists in accordance with the Diagnostic and Statistical Manual of Mental Disorders Fourth Edition (DSM-IV) criteria and the 17-item Hamilton Rating Scale for Depression (HAMD-17). The postmortem intervals between the death of donors and brain collection were less than 40 h and their clinical information is detailed in Supplementary Table 1.

### Microarray

Total blood RNA was isolated using a PAXgene Blood RNA Kit (Qiagen, 762174). The optical density values at 260/280 were approximately 1.9–2.0, and the quality of the RNA was also confirmed with an Agilent 2100 (Agilent Technologies, Santa Clara, CA). The RNA samples underwent reverse transcription to synthesize double-stranded complementary DNA (cDNA), and the cDNA was then transcribed into cRNA in vitro. The cRNA was then reverse-transcribed into cDNA that was then fluorescently labeled and hybridized using a 4 × 180 K lncRNA+mRNA Human Gene Expression Microarray V3.0 (CapitalBio Technology, Beijing, China). The Agilent Feature Extraction (v10.7) software (Santa Clara, CA, USA) was used to analyze the hybridization map and extract the data. The chip fluorescence scanning images were saved as DAT files for analysis by the Agilent G2565CA Microarray Scanner. The data normalization and difference analysis were performed using the Agilent GeneSpring software. Two groups of sample data were analyzed using a t-test analysis to obtain the P-values corrected for multiple testing and the fold change values. The microarray data were validated by qPCR (not published). The expression levels of the genes coding for RNA methylation-modifying enzymes were derived from the microarray data (in-house data).

### Animals

C57BL/6 J male mice (4–6 weeks old) were purchased from Vital River Animal Technology Co., Ltd. (Beijing, China) and bred for 4–5 weeks to perform experiments. C57BL/6 J, *Fto*^*fl/fl*^ mice (Stock No. 027830) were provided by the Jackson Laboratory. These mice possessed loxP sites on either side of exon 3 of *Fto*. Removal of the floxed sequence by Cre recombinase created a null *Fto* allele. Male mice were housed under a 12-h light/dark cycle at 23 ± 2 °C.

### Cell culture

The Neuro-2a cell line, provided by the Cell Resource Center (IBMS, CAMS/PUMC), was cultured in DMEM supplemented with 10% heat-inactivated fetal bovine serum (04-001-1 A, Biological Industries), 100 U/mL penicillin, and 100 μg/mL streptomycin at 37 °C in 5% CO_2_. Plasmids were transfected into Neuro-2a cells using Neofect™ DNA transfection reagent (TF201201, China). siRNA targeting *Ythdf2* (sc-155424, Santa Cruz) was transfected into Neuro-2a cells using RNAi MAX (13778150, Thermo).

### Drug administration

Formoterol (F9552, Sigma) and ICI 118,551 (HY-13951, MedChemExpress) were diluted in saline and administered intraperitoneally at dosages of 2 mg/kg and 10 mg/kg, respectively. To block the effects of formoterol, ICI 118,551 was administered 30 min before formoterol. Fluoxetine (F132, Sigma) diluted in saline was administered intraperitoneally at dosage of 20 mg/kg daily for 14 days.

### Mouse models of depression

#### Unpredictable chronic mild stress (UCMS)

The UCMS model was established in line with a previous work^48^. The animals were subjected to 11 stressors at random for 35 days (three stressors per day from day 1 to day 21 and two stressors per day from day 22 to day 35; the stressors included a hot plate (45 °C/10 min), leaving the lights on overnight, turning the lights off in the daytime, bedding deprivation overnight, crowding (12 h), swimming stress (16 °C/3 min), food and water deprivation (12 h), wet bedding (12 h), an elevated platform (30 min), tilting of the cage at 45 °C (12 h), and restraint stress (1 h).

#### Chronic restraint stress (CRS)

We performed the CRS model by referring to a previous study^49^. Mice were placed in polypropylene conical tubes (50 mL) with pores at the bottom enabling air flow. The animals had no access to food and water during restraint stress. Four hours later, the mice were returned to their home cages. The same stressor was administered once per day at a random time each day, during a total of 21 days.

#### Social defeat stress (SDS)

Social defeat stress exposure was performed in accordance with the published protocol^50^. Briefly, C57BL/6 J mice were placed into a cage in the presence of CD-1 aggressor mice for 5–10 min per day for 10 consecutive days. The C57BL/6 J and CD-1 mice were then separated by a perforated Plexiglas plate for 24 h to prevent any physical contact. Each C57BL/6 J mouse was exposed to different CD-1 mice every day.

#### Behavioral tests

The animals were transported and acclimated to the testing room 1 h before each behavioral test.

#### Forced swimming test (FST)

Mice were individually placed in a beaker (height: 19 cm; diameter: 14 cm) containing 14 cm of water (23 ± 2 °C). The total test duration was 6 min. The process was videotaped, and the immobility time in the last 4 min was scored by an experienced observer blinded to the experimental treatment. Floating or only slight movement to maintain balance was considered as the immobility.

#### Tail suspension test (TST)

The TST was performed using tail suspension hardware (Med Associates, USA). Each mouse was suspended by its tail with a short adhesive tape connected to a load cell that transmitted a signal corresponding to activity. The total test time was 6 min. After setting a low threshold, the duration of immobility was recorded and analyzed by tail suspension software (SOF-821, Med Associates).

#### Sucrose consumption test (SCT)

The mice were acclimatized to 1% sucrose solution for 48 h before the test. Each mouse was individually housed and deprived of water for 12 h. The SCT was performed for 1 h, during which the amount of 1% sucrose solution consumed was recorded.

#### Sucrose preference test (SPT)

The mice were acclimatized to two-bottle drinking (one bottle contained water, and the other contained 1.5% sucrose solution) for 48 h, in which the position of each bottle was switched at 24 h. The mice were then deprived of drinking water for 24 h. The SPT was performed by providing the mice once again with two bottles for 2-h drinking, during which the position of each bottle was switched after 1 h. The ratio of sucrose solution consumed to the total fluid intake in the SPT was determined as the sucrose preference.

#### Social interaction test (SIT)

The SIT was performed in line with a previous report^50^. It consisted of two 2.5-min-long phases: the first one was performed without a CD-1 mouse, and the second one was performed with a CD-1 mouse. The two phases were separated by a pause of 30 s. In both phases, each C57BL/6 J mouse was placed into the rear corner of the open field opposite the wire-mesh compartment. The time spent in the interaction zone (14 cm × 24 cm) was recorded and analyzed by the EthoVision video tracking system (Noldus, the Netherlands). The social interaction ratio was calculated as the ratio between the time spent in the interaction zone in the presence of a CD-1 mouse and the time spent in the absence of the CD-1 mouse. The brains of susceptible and control mice were harvested for gene expression analysis.

#### Novel suppressed feeding test (NSFT)

We performed the NSFT by referring to a previous study^32^. The Mice were deprived of food for 24 h. During the NSFT, and each mouse was placed into the corner of a square chamber (50 cm × 50 cm × 20 cm) opposite a food pellet left in the center of the chamber. The latency to start eating the pellet was recorded. If a mouse did not eat the pellet within 6 min, the latency was recorded as 6 min.

#### Open field test (OFT)

Each mouse was individually placed in one corner of a square Plexiglas box (50 cm × 50 cm × 40 cm) in a brightly lit room, allowing for free exploration of the open field arena for 10 min. The time spent in the center area of the box (12.5 cm × 12.5 cm) and the distance traveled in the box during the testing period were recorded and analyzed by the EthoVision video tracking system (Noldus, the Netherlands).

#### Elevated zero maze test (EZM)

The EZM was performed in a circular elevated maze with two open and two enclosed arms (height: 43.5 cm; diameter: 45 cm; track width: 6.5 cm). Each mouse was individually placed in the cross area of the maze facing the closed arms and allowed to freely explore the maze for 5 min. The time spent in the open arms and the distance traveled in the maze were recorded and analyzed by the EthoVision video tracking system (Noldus, the Netherlands).

#### Vector construction, rAAV packaging and administration

Mouse *Fto* and *Sirt1* cDNAs were cloned into the rAAV expression vector with a CAG promoter. The *Fto* shRNA targeting 5′-GCAGCTGAAATACCCTAAACT -3′ was cloned into the rAAV expression vector with a U6 promoter. AAV-CAG-*Fto*-2A-EGFP (Cat. # AAV2/9-XT071), AAV-CAG-*Sirt1* (Cat. # AAV2/9-XT393), AAV-U6-*Fto* shRNA-CMV-ZsGreen (Cat. # AAV2/9-XT071), AAV-CMV-bGlobin-Cre-eGFP (Cat. # AAV2/9-S0231-9-H50), and AAV-CMV-bGlobin-eGFP-WPRE (Cat. # AAV2/9-S0263-9-H50) were packaged by Taitool Bioscience (Shanghai Taitool Bioscience Co.Ltd., China). The final titer of each rAAV was 3 × 10^12^–4 × 10^12^ vector genome (v.g.)/mL.

#### RNA preparation for quantitative real-time PCR

Total RNA from the hippocampus was extracted using TRIzol (Thermo Fisher Scientific, USA) in line with the manufacturer’s instructions, and the RNA samples were stored at −80 °C. One microgram of total RNA was used to synthesize cDNA by the Transcriptor First Strand cDNA Synthesis kit (04897030001, Roche). Quantitative PCR was performed using FastStart Essential DNA Green Master Mix (06924204001, Roche) on a LightCycler 96 Real-Time System (Roche). The primers used in the qPCR analysis are shown in Supplementary Table 4. Each reaction was performed in triplicate. A fragment of *Gapdh* was amplified as the internal control. Differences in gene expression were calculated by the 2^-ΔΔCT^ method and are presented as the fold change.

#### Western blot analysis

The brain samples were lysed in RIPA lysis buffer with protease inhibitor cocktail (B14001, Bimake) by a tissue homogenizer, followed by ultrasonication and centrifugation. The bicinchoninic acid method was used to determine the concentrations of proteins that were mixed with 5 × Laemmli sample buffer and denatured for 5 min at 95 °C. A total of 30 µg of each sample was separated by 8% sodium dodecyl sulfate-polyacrylamide gel electrophoresis (SDS-PAGE) and then transferred onto nitrocellulose membranes. The membranes were incubated with blocking buffer (TBST buffer containing 5% skim milk powder) for 60 min at room temperature. Next, the membranes were incubated overnight at 4 °C with the primary antibodies. The primary antibodies were as follows: anti-FTO (1:1000, ab92821, Abcam), anti-ADRB2 (1:1000, ab182136, Abcam), anti-SIRT1 (1:1000, 8469 S, Cell Signaling Technology), anti-c-MYC (9402 S, 1:1000, Cell Signaling Technology), and anti-GAPDH (1:10000, GTX100118, GeneTex), HRP-conjugated secondary anti-rabbit (1:5000, GTX213110-01, GeneTex), and HRP-conjugated secondary anti-mouse (1:5000, GTX213111-01, GeneTex). After incubation for 1 h with the corresponding secondary antibodies, the protein bands were detected by chemiluminescence using an ECL reagent.

#### Stereotaxic surgery

Ten-week-old male mice were stereotaxically injected with rAAVs. The mice were anesthetized with 0.7% pentobarbital sodium via intraperitoneally. One microliter of rAAV per side was microinjected bilaterally into the hippocampus (2.2 mm posterior to bregma, ±2.1 mm lateral to midline, and 1.7 mm ventral to bregma) at a rate of 300 nanoliters/min with a Micro4 Syringe Pump Controller (World Precision Instruments, USA). Four weeks later, further experiments were performed. In the experiment of drug infusion into hippocampus, cannulas were implanted bilaterally following the abovementioned coordinates. One week after surgery, formoterol was infused into the hippocampus bilaterally (1 μL each side) at a concentration of 0.4 μg/μL.

#### m^6^A Dot blot

Total RNA in three sample volumes of RNA incubation solution was denatured at 65 °C for 5 min and immediately chilled on ice. The RNA samples were spotted in triplicate onto an Amersham Hybond-N+ membrane (RPN119B, GE Healthcare) with a Bio-Dot Apparatus (#170-6545, Bio-Rad). Next, they were cross-linked to the membrane by ultraviolet light and blocked with a blocking buffer (PBST buffer containing 5% nonfat milk). The membrane was incubated overnight at 4 °C with anti-m^6^A antibody (1:5000, 202003, Synaptic Systems) followed by incubation with an HRP-conjugated goat anti-rabbit IgG (1:5000, sc-2030, Santa Cruz Biotechnology) for one hour at room temperature. Finally, the signal was detected by a chemiluminescent reaction using an ECL reagent.

#### MeRIP-seq

A refined MeRIP-Seq (N6-methyladenosine (m^6^A) RNA immunoprecipitation sequencing) method was performed based on a previously described protocol^51,52^. Each sample was pooled from three mice. The number of samples in each group was as follows: Ctl, *n* = 2; OE, *n* = 2; KD, *n* = 3; Ctl-cKO, *n* = 3; cKO, *n* = 2. RNA was fragmented with the RNA Fragmentation Buffer in the Magna MeRIP™ m^6^A Kit- Transcriptome-wide Profiling of N6-Methyladenosine (17-10499, Millipore). Two microliters of 10 × Fragmentation Buffer were added into 18 µL of mRNA samples. The samples were incubated in the thermal cycler block at 94 °C for 4 min. The samples were taken out from the block and treated with 2 µL of 0.5 M EDTA to stop the reactions. Fragmented RNA was purified by the RNeasy MiniElute Cleanup Kit (QIAGEN, 74204). After enriching for methylated RNA fragments with a specific anti-m^6^A antibody (1:100, ABE572, Millipore), the m6A-seq library was constructed using the SMARTer Stranded Total RNA-Seq Kit-v2 (Pico Input Mammalian, 634413, Takara/Clontech, Japan) and sequenced on Illumina Novaseq 6000.

### Bioinformatics analysis of high-throughput sequencing data

#### Alignment

The sequencing data were analyzed in accordance with the optimized pipeline for refined MeRIP-Seq described in a previous study^52^. The quality control of raw data was performed by FASTQC (version 0.11.5). The clean reads from the m^6^A IP (m^6^A-seq) were aligned to the mouse genome (mm10) using the STAR aligner (version 2.7) with the default parameters after removing reads containing adapters and low-quality reads with Cutadapt (version 1.18). Only uniquely mapped and unduplicated reads remained for the subsequent analysis.

#### m^6^A peak calling and motif analysis

MeTPeak (version 1.1) with the default settings was used for the transcriptome-wide detection of m6A sites in each sample based on the alignment files generated by STAR aligner. The R package Guitar was used to illustrate the distribution of m6A peaks in mRNA^53^. The top 3000 peaks were selected for motif analysis with DREME (version 5.1.1), which used 100-nt-long peak summit-centered sense sequences as an input. The peaks were annotated to the overlapping genomic features and visualized with ChIPseeker (version 1.22.1)^54^ using annotations from TxDb.Mmusculus.UCSC.mm10.knownGene. The peaks were assigned to seven regions, including the promoter, 5′ UTR, 3′ UTR, exon, intron, and downstream and distal intergenic regions.

#### Detection of differential methylation peaks (DMPs)

DMPs were identified in accordance with the pipeline described in the previous study^52^. Four groups were included in our study, including wild-type (WT), KD, cKO and OE mice. The following steps were taken to identify DMPs between three groups based on the expression levels in *Fto*-modified and control samples (WT mice). First, the overlapping m^6^A peaks from two samples were defined as common peaks. The common peaks between samples from the same group were merged and combined with the common peaks from another group to form the m^6^A union peaks reference list. The enrichment score of each sample for each peak was obtained by dividing the read counts for IP by the input. High confidence DMPs meeting the criteria of an average enrichment score higher than 6 in the groups and a log2 fold change >1 or <−1 were selected for further analysis. The DMPs were annotated in accordance with the genomic features with ChIPseeker (version 1.22.1). Functional enrichment analysis of the gene ontology (GO) and pathways was performed using ChIPseeker (version 1.22.1) to identify the predominant biological functions of the annotated genes nearest to the DMPs. Fisher’s exact test was applied to identify the significant GO categories and pathways, and FDR correction was performed with a threshold of 0.05. Input RNA before immunoprecipitation was applied to RNA-seq in parallel.

#### Gene-specific m^6^A qPCR

The Magna MeRIP m^6^A kit (17-10499, Millipore) was used to perform m^6^A RNA immunoprecipitation in line with the manufacturer’s instructions. A QuantiTect SYBR Green RT-PCR Kit (204243, Qiagen) was used to perform reverse transcription and real-time qPCR. The relative expression of the m^6^A-modified target gene was determined as the Cq value of the m^6^A IP portion divided by the Cq value of the input portion.

#### RNA stability

Neuro-2a cells cultured in 6-well plates at 80–90% confluence were transfected with 2 µg of *Fto*-shRNA, FTO overexpression, or control vector. Forty-eight hours later, the cells were treated with 5 μg/ml actinomycin D (A9415, Sigma) and harvested at 0, 3 and 6 h. Total RNA was extracted using TRIzol, and 1 μg of total RNA was used for reverse transcription and qPCR.

#### Luciferase assay

In *Adrb2* 3' UTR fragment, there are a total of four possible m^6^A modification sites, predicted by the “DRACH” motif (D = A/G, R = A/G, H = A/C/T) from the MeRIP-analysis^22^. We generated a mutant fragment by replacing “A” into “T” in all the four possible m^6^A modification sites. Wild type and mutant *Adrb2*-3' UTR were synthetized and inserted into pMIR-GLO vectors (Tsingke). For dual-luciferase reporter assays, 50 ng pMIR-GLO vectors and 50 ng pcDNA3.1_*Fto* or pcDNA3.1_mut*Fto* were co-transfected into HEK293 cells in 24-well plates. The mutant FTO-expressing vector contains the full-length *Fto* coding region with mutations at H231A and D233A, which results in complete loss of m^6^A demethylation activity^55,56^. Relative luciferase activities were assessed by Dual-Luciferase Reporter Assay System (Promega) after 48 h transfection.

1. Adrb2-3' UTR with wild-type m^6^A sites
  TCTAGATAGTGTCCTGTCAAGGAGGGGTCTTAGAGAGTAGAAAGCCTGTATTACAGTGGCGAGTCATTTGTACTACAGTTCCTTCCTTGGGAGTCAACGCTAAGGCTAGGCACAGTACCTTGACAGTTCACAAAGCCTTCCATGCCTGGGGGATCCTCACACAGCAGTTCCTGTTCTCTCTCCTGCCCCAGCTGACAAGTGTTTGGCTCCCCTGTGTAGTCCGTTCTGCCGTTGCTATTGCTAGAGTAGCCGTTCCCATAGGTTTTCGAAGA**AGACC**TGCGAAGGCACAGAAGCTCTTGAAAGGCAATCCTGAAATCT**GGACT**CCGACAGTAGATAAGAGGATTGAAGGCAGAGTTGACGTAGCCCAACCAGTTAAGGAGGATGTAAACTTCCTTAGGGATGAGGTTGTCCCTGATAACGTGCACGATATTGACAATGAAGAAGGGCAGCCAGCAGAGGGTGAATGTGCCCATGATGATGCCTAAAGTCTTGAGGGCTTTGTGCTCTTTCAAGCA**GAACT**TGGA**GGACC**TTCGGAGTCCGTGGCCGCTCCGCCCATCCTGCTCCACCTGGCTGAGGTTTTGGGCGTGGAATCTTCCTTCGCGGCCGC
2. Adrb2-3' UTR with mutant m^6^A sites
  TCTAGATAGTGTCCTGTCAAGGAGGGGTCTTAGAGAGTAGAAAGCCTGTATTACAGTGGCGAGTCATTTGTACTACAGTTCCTTCCTTGGGAGTCAACGCTAAGGCTAGGCACAGTACCTTGACAGTTCACAAAGCCTTCCATGCCTGGGGGATCCTCACACAGCAGTTCCTGTTCTCTCTCCTGCCCCAGCTGACAAGTGTTTGGCTCCCCTGTGTAGTCCGTTCTGCCGTTGCTATTGCTAGAGTAGCCGTTCCCATAGGTTTTCGAAGA**AGTCC**TGCGAAGGCACAGAAGCTCTTGAAAGGCAATCCTGAAATCT**GGTCT**CCGACAGTAGATAAGAGGATTGAAGGCAGAGTTGACGTAGCCCAACCAGTTAAGGAGGATGTAAACTTCCTTAGGGATGAGGTTGTCCCTGATAACGTGCACGATATTGACAATGAAGAAGGGCAGCCAGCAGAGGGTGAATGTGCCCATGATGATGCCTAAAGTCTTGAGGGCTTTGTGCTCTTTCAAGCA**GATCT**TGGA**GGTCC**TTCGGAGTCCGTGGCCGCTCCGCCCATCCTGCTCCACCTGGCTGAGGTTTTGGGCGTGGAATCTTCCTTCGCGGCCGC

#### Golgi staining

The mice were anesthetized with chloral hydrate (10%, wt/vol, i.p.) and sequentially perfused transcardially with saline. The whole brain was freshly harvested and immediately immersed in one well of a 12-well plate containing 5 mL of solution A (superGolgi Kit, Cat. # 003010, Bioenno Tech, LLC). In accordance with the manufacturer’s instructions, the solution was renewed two days later, and the impregnation continued for another nine days. Next, the brain was transferred to postimpregnation buffer for two days with renewal of the solution after one day of immersion. Sections (100 μm) in thickness were cut using a vibratome (VT1200S, Leica). The sections were mounted on adhesive microscope slides (SUPERFROST® PLUS, Cat. # 4951 PLUS-100E, Thermo Scientific). The staining time in solutions C and D was 20 min. Then, the sections were dehydrated in 100% ethanol for 7 min four times each, which was followed by vitrification with xylene for 8 min 10 times each. Finally, the sections were coverslipped with neutral resins. For spine density measurement, we imaged one terminal dendrite from the CA1 region of the hippocampus using a 100 × objective on a microscope (DM6 B, Leica). A Z-stack of images with an interval of 0.22 μm was acquired for each dendritic segment, which was followed by an extended depth of field algorithm calculation to obtain in focus images using LAS X software (Leica Application Suite X, Leica). The diameters of the selected segments were comparable. A total of 120 segments from 20 mouse brains (six segments per animal) were used to determine the spine number. The dendritic spines, including thin, stubby, and mushroom types, within a 15-μm length segment were counted manually by an investigator blinded to the experimental treatments.

#### ChIP-qPCR

SimpleChIP Plus Enzymatic chromatin IP kit (Cell Signaling Technology; #9005) was used to perform ChIP assay. N2A cells (10^6^ per ChIP sample) were cross-linked with 1% formaldehyde and incubated at 37 °C for 10 min, then stopped by glycine. Micrococcal nuclease was added into cells to digest nuclei, and the lysates were sonicated to disrupt nuclear membranes. The samples were incubated with 5 µg c-MYC antibodies overnight at 4 °C, and then immunoprecipitated with protein G magnetic beads for 2 h. Eluted DNA was used for ChIP–qPCR analysis with primers listed in Supplementary Table 4.

#### Statistical analysis

Statistical analysis was conducted using the Prism software (GraphPad Prism 8). Two-tailed student’s t test was used for comparisons between the two groups, and one-way or two-way analysis of variance (ANOVA) was performed for more than two groups. *p* < 0.05 was considered statistically significant. All the data were exported into Adobe Photoshop CS6 for the preparation of figures. All data are shown as the mean ± standard error of mean (s.e.m).

### Reporting summary

Further information on research design is available in the Nature Research Reporting Summary linked to this article.

## Supplementary information

Supplementary Information

Reporting summary

## Data Availability

The MeRIP-seq data generated in this study have been deposited in the Genome Sequence Archive under accession code CRA005192 (https://ngdc.cncb.ac.cn/gsa/)^57,58^. Uncropped and unprocessed scans are supplied in the Source data. Source data are provided as a Source Data.xlsx. Source data are provided with this paper.

## Source data

Source Data
