# Supplementary material for: Fat mass and obesity-associated protein regulates RNA methylation associated with depression-like behavior in mice
Source: Nat Commun. 2021 Nov 26;12:6937. doi: 10.1038/s41467-021-27044-7 (PMC8626436; doi:10.1038/s41467-021-27044-7)
Supplement: Supplementary file 1 — Supplementary Information [file 41467_2021_27044_MOESM1_ESM.pdf]

Supplementary Information

Liu et al.

Fat mass and obesity-associated protein regulates RNA methylation associated with depression-like behavior in mice.

Supplementary Figure 1.

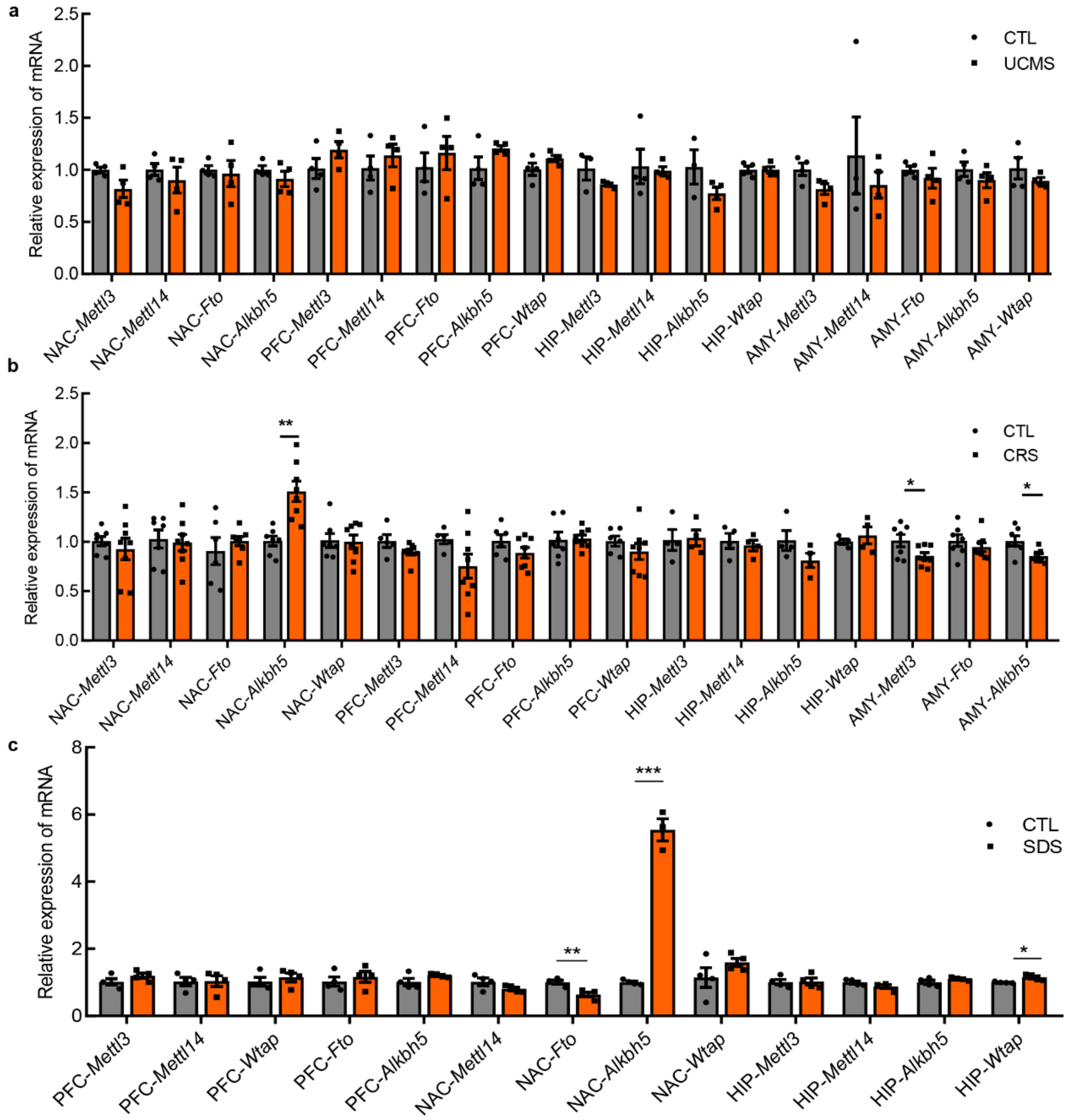

Supplementary Figure 1. mRNA expression of genes encoding m^{6}A-modifying enzymes in emotion-regulating brain regions of mouse models of depression

(a) mRNA expression in the UCMS model. NAC, nucleus accumbens; PFC, prefrontal cortex; HIP, hippocampus; AMY, amygdala, n = 4 per group except for HIP-*Mettl3* CTL**,** HIP-*Alkbh5***,** n = 3. **(b)** mRNA expression in the CRS model, NAC-*Mettl3*, NAC-*Mettl14*, NAC-*Alkbh5*, NAC-*Wtap*, PFC-*Alkbh5*, AMY-*Mettl3*, AMY-*Fto*, AMY-*Alkbh5* CTL and NAC-*Fto*, AMY-*Alkbh5* CRS, n = 7; PFC-*Mettl3* and PFC-*Mettl14*, n

= 5; NAC-*Fto*, PFC-*Fto* and PFC-*Wtap* CTL, n = 6; HIP-*Mettl3*, HIP-*Mettl14*, HIP-*Alkbh5*, HIP-*Wtap* CTL and CRS, n = 4; NAC-*Mettl3*, NAC-*Mettl14*, NAC-*Alkbh5*, NAC-*Wtap*, PFC-*Mettl3*, PFC-*Mettl14*, PFC-*Fto*, PFC-*Alkbh5*, PFC-*Wtap*, Amy-*Mettl3* and AMY-*Fto* CRS, n = 8.. NAC-*Alkbh5*, p = 0.001004; AMY-*Mettl3*, p = 0.042111; AMY-*Alkbh5*, p = 0.019478. **(c)** mRNA expression in the SDS model. n = 4 per group except for NAC-*Alkbh5* n = 3. NAC-*Fto*, p = 0.006863; NAC-*Alkbh5*, p = 0.000016; HIP-*Wtap*, p = 0.016705. *p < 0.05, **p < 0.01, ***p < 0.001. Two-tailed Student's t-test. Error bars represent s.e.m. Source data are provided as a Source Data file.

Supplementary Figure 2.

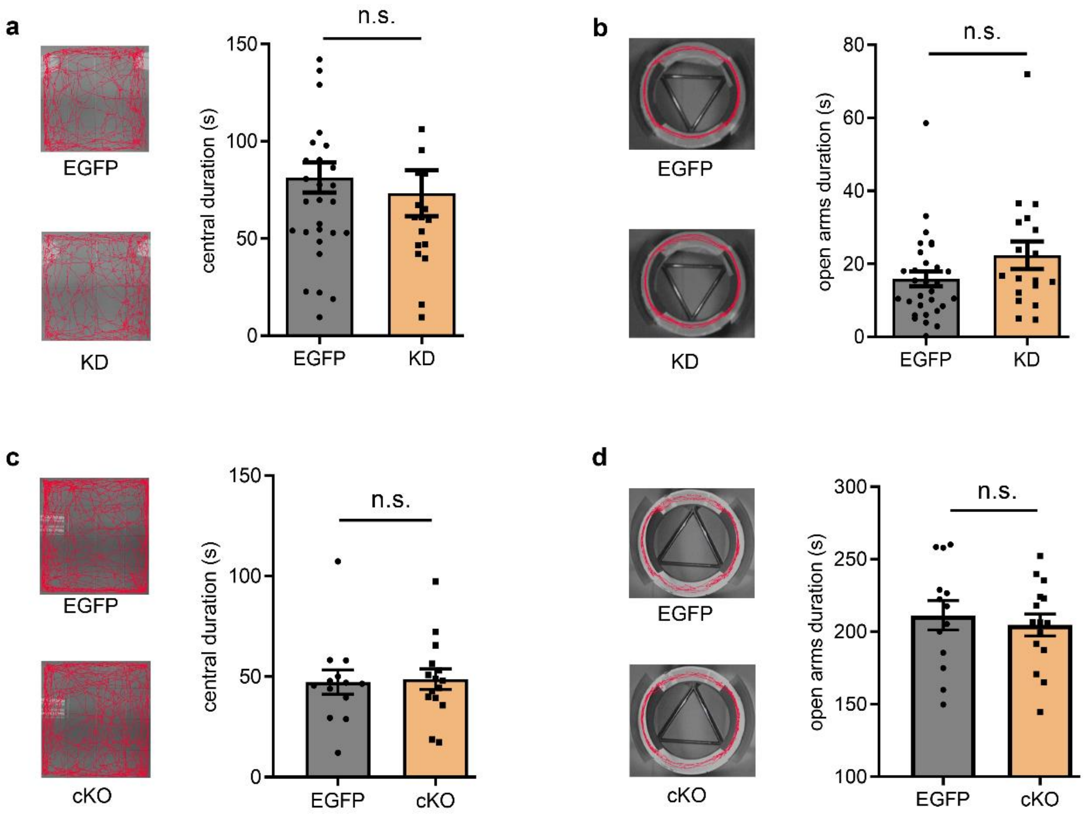

Supplementary Figure 2. Suppression of the expression of FTO in the hippocampus does not affect anxiety-like behaviors

(a) Representative images of movement tracks and the central duration in the open field test (OFT) for KD mice. Ctl, n = 31; KD, n = 18. **(b)** Representative images of movement tracks and the duration in the open arms in the elevated zero maze test (EZM) for KD mice. Ctl, n = 31; KD, n = 18. **(c)** Representative images of movement tracks and the central duration in the open field test (OFT) for cKO mice. Ctl, n = 13; cKO, n = 15. **(d)** Representative images of movement tracks and the duration in the open arms in the elevated zero maze test (EZM) in cKO mice. Ctl, n = 13; cKO, n = 15. n.s., no significant. Two-tailed Student's t-test. Error bars represent s.e.m. Source data are provided as a Source Data file.

Supplementary Figure 3.

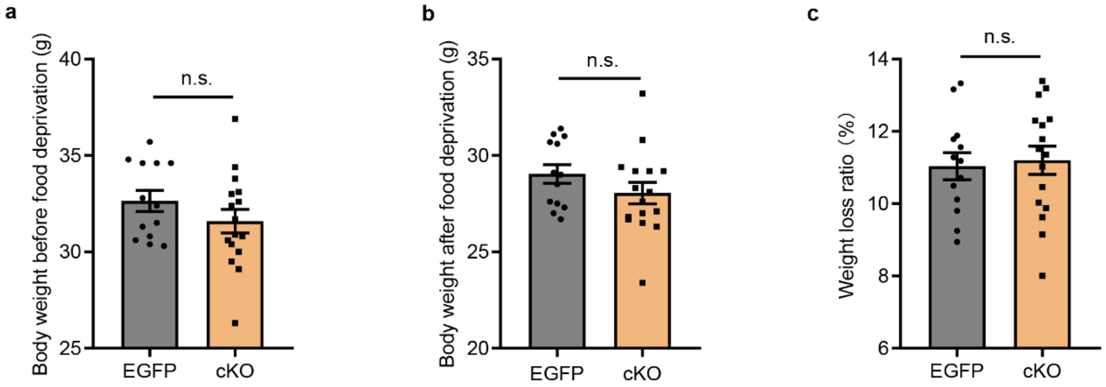

Supplementary Figure 3. *Fto* knockout in the hippocampus does not affect body weight or weight loss in response to food deprivation

(a) Body weight of cKO and control mice before food deprivation. **(b)** Body weight of cKO and control mice after 24 h of food deprivation. **(c)** Weight loss ratio of cKO and control mice in response to 24 h of food deprivation. EGFP, n = 13; cKO, n = 16. n.s., no significant. Two-tailed Student’s t-test. Error bars represent s.e.m. Source data are provided as a Source Data file.

Supplementary Figure 4.

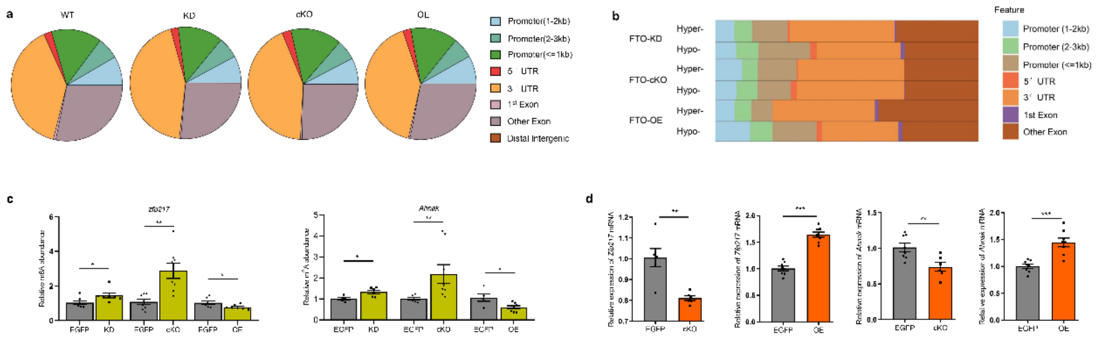

pplementary Figure 4. Analysis of m^{6}A-modified genes regulated by FTO

(a) Annotation of m^{6}A peaks detected in different samples. **(b)** Annotation of differentially methylated peaks detected in KD, cKO, and OE mice. **(c)** Gene-specific m6A qPCR assay used to verify the m^{6}A levels in the *Zfp217* and *Ahnak* mRNA, which are the identified targets modified by FTO. *Zfp217*, n = 7, 7, 8, 8, 7, 7 from left to right; *Ahnak*, n = 5, 6, 6, 8, 5, 8 from left to right. *Zfp217*, EGFP vs. KD, p = 0.0353, EGFP vs. cKO, p = 0.0011; EGFP vs. OE, p = 0.0432. *Ahnak*, EGFP vs. KD, p = 0.0159; EGFP vs. cKO, p = 0.0047; EGFP vs. OE, p = 0.0166. **(d)** Expression of the *Zfp217* and *Ahnak* mRNAs in the hippocampi of cKO and OE mice. *Zfp217*, EGFP vs. cKO, n = 6 per group, p = 0.0043; EGFP vs. OE, n = 8, p < 0.0001; *Ahnak*, EGFP, n = 8; cKO, n = 6, EGFP vs. cKO, p = 0.0084; EGFP vs. OE, n = 8, p = 0.0002. *p < 0.05, **p < 0.01, ***p < 0.001. Two-tailed Student's t-test. Error bars represent s.e.m. Source data are provided as a Source Data file.

Supplementary Figure 5.

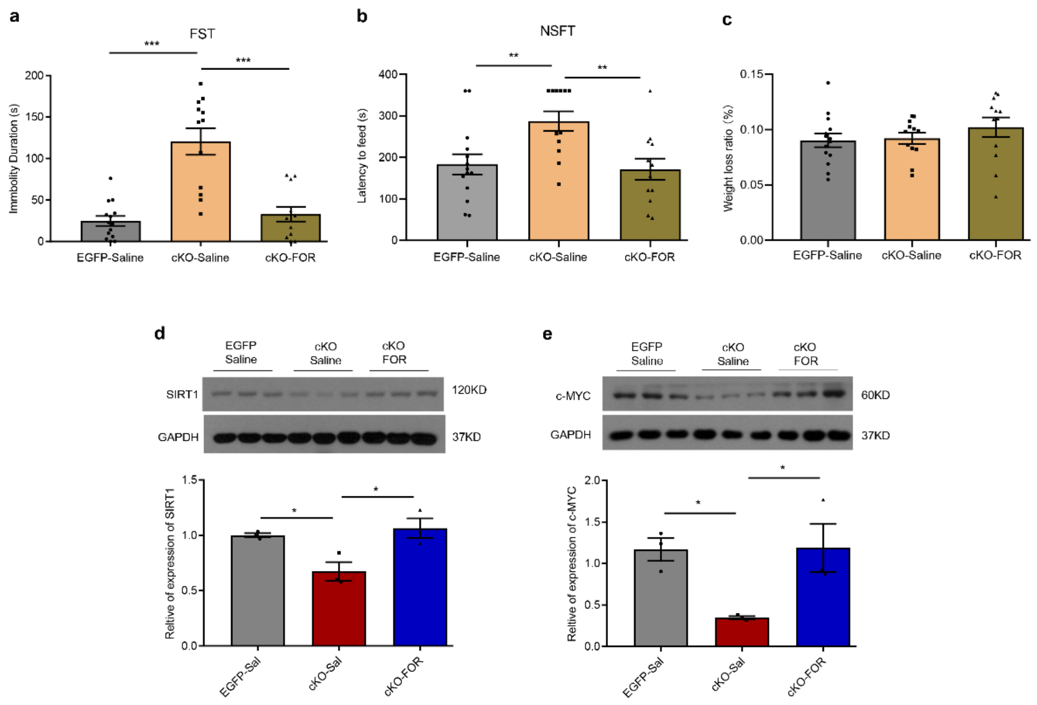

Supplementary Figure 5. ADRB2 stimulation in the hippocampus rescues depression-like behaviors and c-MYC/SIRT1 protein expression caused by hippocampal FTO deficiency

(a-c) FST, NSFT and weight loss ratio after infusion with FOR in the hippocampus. EGFP-Sal, n = 14; cKO-Sal, n = 12; cKO + FOR, n = 12. (a) EGFP-Sal vs. cKO-Sal, $p < 0.0001$; cKO-Sal vs. cKO+FOR, $p < 0.0001$. (b) EGFP-Sal vs. cKO-Sal, p = 0.0097; cKO-Sal vs. cKO+FOR, p = 0.0054. **(d, e)** SIRT1 and c-MYC expression in the hippocampi of *Fto*-cKO mice treated with FOR, n = 3 (d) EGFP-Sal vs. cKO-Sal, p = 0.0357; cKO-Sal vs. cKO+FOR, p = 0.0166. (e) EGFP-Sal vs. cKO-Sal, p = 0.0404; cKO-Sal vs. Cko+FOR, p = 0.0366. Source data are provided as a Source Data file.

Supplementary Table 1. Information of postmortem human brains

	Sex	Age (years)	Autopsy delay (h)
Control	2 Female<br>1 Male	81-87	6-17.6
Depression	2 Female<br>1 Male	72-87	6-39.0

Supplementary Table 2. Summary of the m6A-seq and RNA-seq of samples

Sample	RNA-seq		m6A-seq		Peaks	Genes	Common Peaks	Genes Count
	Total reads	Aligned Reads	Total reads	Aligned Reads				
WT-1	58.25M	30.28M (52.00%)	64.48M	53.26M (82.59%)	11003	5106	10162	4630
WT-2	50.83M	27.06M (53.25%)	55.95M	48.23M (86.19%)	15036	6782		
FTO-OE1	55.25M	25.25M (46.97%)	54.58M	45.04M (82.53%)	13543	5891	11531	4730
FTO-OE2	70.12M	33.41M (47.66%)	51.30M	42.20M (86.47%)	12681	5486		
FTO-KD1	53.55M	28.71M (53.62%)	58.71M	48.42M (82.47%)	14716	6286	13250	5043
FTO-KD2	61.92M	33.43M (53.98%)	48.44M	41.57M (85.82%)	15119	6801		
FTO-KD3	50.62M	29.22M (57.73%)	47.01M	39.71M (84.47%)	13754	6042		
WT-4	126.67M	95.55M(75.44%)	60.60M	56.90M (93.89%)	14420	6625	16959	5498
WT-5	134.41M	102.73M(76.43%)	76.53M	71.86M (93.89%)	15670	7052		
WT-6	135.57M	103.37M(76.25%)	64.64M	60.80M (94.06%)	14625	6625		
FTO-cKO1	144.58M	108.02M(74.72%)	60.15M	56.19M (93.42%)	15558	6889	14833	6072
FTO-cKO2	142.66M	107.61M(75.44%)	64.58M	60.04M (92.97%)	15064	6820		

M million; WT wild type; FTO-cKO FTO-conditional knockout; FTO-OE FTO-overexpression; FTO-KD FTO-knockdown.

Supplementary Table 3. Log2(fold change) of potential targets of FTO

Gene	FTO-KD	FTO-cKO	FTO-OE
Gm12992	2.50	6.45	-9.74
Zim3	5.73	5.64	-8.48
Gcnt1	1.36	4.10	-3.75
Zfp217	1.56	5.67	-2.77
5830417I10Rik	1.11	1.78	-2.57
Zscan29	2.39	1.15	-1.94
Pcdhb11	1.31	1.72	-1.88
Adrb2	1.33	7.52	-1.81
Ago4	1.30	3.47	-1.74
Dnase1	7.77	7.72	-1.70
Abi3	1.38	1.16	-1.60
Ntn3	1.03	1.06	-1.56
Zfp599	1.45	1.85	-1.56
Itpkc	1.40	3.80	-1.53
Ahnak	1.20	1.37	-1.50
Celsr1	1.84	1.44	-1.49
Nrros	1.66	2.41	-1.47
Arsj	2.69	2.24	-1.47
Pcdhb4	1.19	1.00	-1.47
Fam124a	1.12	5.23	-1.40
Dse	1.17	8.21	-1.39
Zfp551	1.23	1.18	-1.38
Pnma1	2.21	1.84	-1.37
Amer1	1.03	1.57	-1.36
9130024F11Rik	1.31	1.47	-1.31
Gm9899	1.78	1.46	-1.26
Gm6981	1.81	1.50	-1.25
C1ra	2.33	1.08	-1.23
Ankrd34c	2.12	6.83	-1.15
Bmf	2.00	6.55	-1.14
Nfatc2	1.75	6.96	-1.13
Kif26a	1.38	1.51	-1.07
Zfp786	1.08	1.82	-1.06
Fndc9	1.76	6.03	-1.04

Supplementary Table 4. Primers

Gene name	Forward Primer	Reverse Primer
Mettl3	GGACTCTGGGCACTTGGATTT	ATCAGTGGGCAAGGTCAAGG
Mettl14	TGGGAGAGTATGCTTGCGAA	CACGGTTCCTTTGATCCCCA
Wtap	TCTTGTCATGCGGCTAGCAA	GCGTAAACTTCCAGGCACTC
Fto	CTATAGCTGCGAAGGCTCTG	TAGCAGTCTCCCTGGTGAAGA
Alkbh5	CCCCATCCACATCTTCGAGC	ATCAGCAGCATACCCACTGAG
hMETTL3	TTCGAGAGGTGTCAGGGCT	CGGAAGGTTGGAGACAATGC
hMETTL14	CAGAGAACAAAGGAACACTGCC	TTGGTCCAACTGTGAGCCAG
hWTAP	ACAACAGCAGGAGTCTGCAC	GATCTCAGTTGGGCAACGCT
hFTO	GCACACGGGAAGGAGATGTT	ATGTCATCAGGATGGGCCAC
hALKBH5	CTATTCGGGTGTCGGAACCA	GGGTGCATCTAATCTTGTCTTCT
Gapdh	TGTGTCCGTCGTGGATCTGA	TTGCTGTTGAAGTCGCAGGAG
hGAPDH	CGGAGTCAACGGATTTGGTC	TGGGTGGAATCATATTGGAACAT
Adrb2	ATGTCGGTTATCGTCCTGGC	GGTTTGTAGTCGCTCGAACTTG
hADRB2	TGGTGTGGATTGTGTCAGGC	GGCTTGGTTCGTGAAGAAGTC
Sirt1	ATGTCGGTTATCGTCCTGGC	GGTTTGTAGTCGCTCGAACTTG
Zfp217	AGTCCCCTTGATAAAGCTCTGG	GCACCCATACGTGAAGGAGTC
Ahnak	CCACCCCAACTGGGACTTTG	CACTCCCCTGTAACTTGCCTG
Adrb2 Gene-specific m6A qPCR	GGGCTTTGTGCTCTTTCAA	TCTATTCCCGGGTCTTCCA
Zfp217 Gene-specific m6A qPCR	ATACTCAACAGAACCGTTGCA	GTCACACAGGTCACAACCCAT
Ahnak Gene-specific m6A qPCR	GAGCCTGCCTAAGGCTAACA	GGCCATGAACATCCACATCT
Sirt1 E1	AGACAGGGAGGGATGGATG	AGCCTGGCTATGTCCACAAT
Sirt1 E2	CCTCACTGCTCCCACAGAG	TCCGTGGAAGTGTACCTTGG
