# Supplementary material for: Fat mass and obesity-associated protein regulates RNA methylation associated with depression-like behavior in mice
Source: Nat Commun. 2021 Nov 26;12:6937. doi: 10.1038/s41467-021-27044-7 (PMC8626436; doi:10.1038/s41467-021-27044-7)
Supplement: Supplementary file 2 — Reporting summary [file 41467_2021_27044_MOESM2_ESM.pdf]

nature research

Corresponding author(s): Qi Xu

Last updated by author(s): Oct 18, 2021

Reporting Summary

Nature Research wishes to improve the reproducibility of the work that we publish. This form provides structure for consistency and transparency in reporting. For further information on Nature Research policies, see our Editorial Policies and the Editorial Policy Checklist.

Statistics

For all statistical analyses, confirm that the following items are present in the figure legend, table legend, main text, or Methods section.

n/a	Confirmed	
☐	☒	The exact sample size (*n*) for each experimental group/condition, given as a discrete number and unit of measurement
☐	☒	A statement on whether measurements were taken from distinct samples or whether the same sample was measured repeatedly
☐	☒	The statistical test(s) used AND whether they are one- or two-sided<br>*Only common tests should be described solely by name; describe more complex techniques in the Methods section.*
☒	☐	A description of all covariates tested
☐	☒	A description of any assumptions or corrections, such as tests of normality and adjustment for multiple comparisons
☐	☒	A full description of the statistical parameters including central tendency (e.g. means) or other basic estimates (e.g. regression coefficient) AND variation (e.g. standard deviation) or associated estimates of uncertainty (e.g. confidence intervals)
☐	☒	For null hypothesis testing, the test statistic (e.g. *F*, *t*, *r*) with confidence intervals, effect sizes, degrees of freedom and *P* value noted<br>*Give P values as exact values whenever suitable.*
☒	☐	For Bayesian analysis, information on the choice of priors and Markov chain Monte Carlo settings
☒	☐	For hierarchical and complex designs, identification of the appropriate level for tests and full reporting of outcomes
☒	☐	Estimates of effect sizes (e.g. Cohen's *d*, Pearson's *r*), indicating how they were calculated

Our web collection on statistics for biologists contains articles on many of the points above.

Software and code

Policy information about availability of computer code

Data collection	Tail suspension software was used for tail suspension test (SOF-821, Med Associates); EthoVision video tracking system (Noldus, the Netherlands) was used for open field and elevated zero maze test; microscope (DM6 B, Leica) was used for golgi staining.
Data analysis	GradPad 8.0.1 was mostly used for statistical analysis; Image J (1.4.3.67)was used for western blot analysis. Quality control was conducted using FASTQC (version 0.11.5), cutadapt (version 1.18). Alignment was conducted using STAR aligner (version 2.7). Peak calling and annotation analysis were conducted using MeTPeak (version 1.1),ChIPseeker (version 1.22.1). Motif analysis was conducted using DREME (version 5.1.1), Guitar (version 3.11) .

For manuscripts utilizing custom algorithms or software that are central to the research but not yet described in published literature, software must be made available to editors and reviewers. We strongly encourage code deposition in a community repository (e.g. GitHub). See the Nature Research guidelines for submitting code & software for further information.

Data

Policy information about availability of data

All manuscripts must include a data availability statement. This statement should provide the following information, where applicable:

- Accession codes, unique identifiers, or web links for publicly available datasets
- A list of figures that have associated raw data
- A description of any restrictions on data availability

Raw sequencing data is deposited to the public data repository (Genome Sequence Archive), and the accession ID is included in Data Availability statement. The data for gels and graphs in this study are available in the sourse data, other data is available from the corresponding authors based on reasonable request.

Field-specific reporting

Please select the one below that is the best fit for your research. If you are not sure, read the appropriate sections before making your selection.

☒ Life sciences ☐ Behavioural & social sciences ☐ Ecological, evolutionary & environmental sciences

For a reference copy of the document with all sections, see nature.com/documents/nr-reporting-summary-flat.pdf

Life sciences study design

All studies must disclose on these points even when the disclosure is negative.

Sample size	No statistical methods were applied to determine the sample size, the sample size are similar to those reported in previous literature.
Data exclusions	No data were excluded.
Replication	All experiments were performed at least two replicates.
Randomization	The mice used in all experiments were assigned randomly to either control or experimental groups.
Blinding	All behavioral data analysis in this study were carried out blind to prevent any bias.

Behavioural & social sciences study design

All studies must disclose on these points even when the disclosure is negative.

Study description	*Briefly describe the study type including whether data are quantitative, qualitative, or mixed-methods (e.g. qualitative cross-sectional, quantitative experimental, mixed-methods case study).*
Research sample	*State the research sample (e.g. Harvard university undergraduates, villagers in rural India) and provide relevant demographic information (e.g. age, sex) and indicate whether the sample is representative. Provide a rationale for the study sample chosen. For studies involving existing datasets, please describe the dataset and source.*
Sampling strategy	*Describe the sampling procedure (e.g. random, snowball, stratified, convenience). Describe the statistical methods that were used to predetermine sample size OR if no sample-size calculation was performed, describe how sample sizes were chosen and provide a rationale for why these sample sizes are sufficient. For qualitative data, please indicate whether data saturation was considered, and what criteria were used to decide that no further sampling was needed.*
Data collection	*Provide details about the data collection procedure, including the instruments or devices used to record the data (e.g. pen and paper, computer, eye tracker, video or audio equipment) whether anyone was present besides the participant(s) and the researcher, and whether the researcher was blind to experimental condition and/or the study hypothesis during data collection.*
Timing	*Indicate the start and stop dates of data collection. If there is a gap between collection periods, state the dates for each sample cohort.*
Data exclusions	*If no data were excluded from the analyses, state so OR if data were excluded, provide the exact number of exclusions and the rationale behind them, indicating whether exclusion criteria were pre-established.*
Non-participation	*State how many participants dropped out/declined participation and the reason(s) given OR provide response rate OR state that no participants dropped out/declined participation.*
Randomization	*If participants were not allocated into experimental groups, state so OR describe how participants were allocated to groups, and if allocation was not random, describe how covariates were controlled.*

Ecological, evolutionary & environmental sciences study design

All studies must disclose on these points even when the disclosure is negative.

Study description	*Briefly describe the study. For quantitative data include treatment factors and interactions, design structure (e.g. factorial, nested, hierarchical), nature and number of experimental units and replicates.*
Research sample	*Describe the research sample (e.g. a group of tagged Passer domesticus, all Stenocereus thurberi within Organ Pipe Cactus National Monument), and provide a rationale for the sample choice. When relevant, describe the organism taxa, source, sex, age range and any manipulations. State what population the sample is meant to represent when applicable. For studies involving existing datasets, describe the data and its source.*

Sampling strategy	*Note the sampling procedure. Describe the statistical methods that were used to predetermine sample size OR if no sample-size calculation was performed, describe how sample sizes were chosen and provide a rationale for why these sample sizes are sufficient.*
Data collection	*Describe the data collection procedure, including who recorded the data and how.*
Timing and spatial scale	*Indicate the start and stop dates of data collection, noting the frequency and periodicity of sampling and providing a rationale for these choices. If there is a gap between collection periods, state the dates for each sample cohort. Specify the spatial scale from which the data are taken*
Data exclusions	*If no data were excluded from the analyses, state so OR if data were excluded, describe the exclusions and the rationale behind them, indicating whether exclusion criteria were pre-established.*
Reproducibility	*Describe the measures taken to verify the reproducibility of experimental findings. For each experiment, note whether any attempts to repeat the experiment failed OR state that all attempts to repeat the experiment were successful.*
Randomization	*Describe how samples/organisms/participants were allocated into groups. If allocation was not random, describe how covariates were controlled. If this is not relevant to your study, explain why.*
Blinding	*Describe the extent of blinding used during data acquisition and analysis. If blinding was not possible, describe why OR explain why blinding was not relevant to your study.*

Did the study involve field work? ☐ Yes ☐ No

Field work, collection and transport

Field conditions	*Describe the study conditions for field work, providing relevant parameters (e.g. temperature, rainfall).*
Location	*State the location of the sampling or experiment, providing relevant parameters (e.g. latitude and longitude, elevation, water depth).*
Access & import/export	*Describe the efforts you have made to access habitats and to collect and import/export your samples in a responsible manner and in compliance with local, national and international laws, noting any permits that were obtained (give the name of the issuing authority, the date of issue, and any identifying information).*
Disturbance	*Describe any disturbance caused by the study and how it was minimized.*

Reporting for specific materials, systems and methods

We require information from authors about some types of materials, experimental systems and methods used in many studies. Here, indicate whether each material, system or method listed is relevant to your study. If you are not sure if a list item applies to your research, read the appropriate section before selecting a response.

Materials & experimental systems

n/a	Involved in the study	
☐	☒	Antibodies
☐	☒	Eukaryotic cell lines
☒	☐	Palaeontology and archaeology
☐	☒	Animals and other organisms
☐	☒	Human research participants
☒	☐	Clinical data
☒	☐	Dual use research of concern

Methods

n/a	Involved in the study	
☒	☐	ChIP-seq
☒	☐	Flow cytometry
☒	☐	MRI-based neuroimaging

Antibodies

Antibodies used	Antibodies for western blot : anti-FTO (Abcam, Cat ab92821, 1:1000,), anti-ADRB2 (Abcam, ab182136,1:1000), anti-SIRT1 (Cell Signaling Technology, 8469S, 1:1000), anti-c-MYC (Cell Signaling Technology, 9402S, 1:1000), anti-GAPDH (1:10000, GTX100118, GeneTex), anti Rabbit (GeneTex, GTX213110-01, 1:5000,), and anti-Mouse (GeneTex, GTX213111-01 ,1:5000); antibodies for m6A blot : anti-m6A antibody (Synaptic Systems, 202003, 1:5000); antibodies for m6A blot : anti-m6A antibody (Synaptic Systems, 202003, 1:5000); antibodies for MeRIP-seq: anti-m6A antibody (Millipore, ABE572, 1:100); antibodies for gene-specific m6A qPCR: anti-m6A antibody (Synaptic Systems, 202003, 1:1000).
Validation	All antibodies used in our study were validated on the manufacturer's website.<br>anti-FTO (ab92821, Abcam) https://www.abcam.cn/fto-antibody-5-2h10-ab92821.html<br>anti-Adrb2 (ab182136, Abcam) https://www.abcam.cn/beta-2-adrenergic-receptor-antibody-epr707n-ab182136.html<br>anti-Sirt1 (8469S, Cell Signaling Technology) https://www.cellsignal.cn/products/primary-antibodies/sirt1-1f3-mouse-mab/8469?N=4294956287&Ntt=sirt1&fromPage=plp<br>anti-c-Myc (9402S, , Cell Signaling Technology) https://www.cellsignal.cn/products/primary-antibodies/c-myc-antibody/9402?<br>anti-GAPDH (GTX100118, GeneTex) https://www.genetex.cn/Product/Detail/GAPDH-antibody/GTX100118

anti-m6A antibody (202003, Synaptic Systems) https://www.sysy.com/product/202003#list
anti-m6A antibody (ABE572, Millipore) https://www.merckmillipore.com/CN/zh/product/Anti-N6-methyladenosine-m6A-Antibody,MM_NF-ABE572
anti Rabbit (GTX213110-01, GeneTex) https://www.genetex.cn/Product/Detail/Goat-Anti-Rabbit-IgG-antibody-HRP/GTX213110-01
anti-Mouse (GTX213111-01 , GeneTex) https://www.genetex.cn/Product?category=0&keyword=anti+mouse&page=1

Eukaryotic cell lines

Policy information about cell lines

Cell line source(s)	The N2A cells used in this study were purchased from DBPR (manuscript NCOMMS-21-04052A).
Authentication	The Neuro-2a cell line was commercially available and has not been authenticated
Mycoplasma contamination	N2A cells tested negative for mycoplasma contamination.
Commonly misidentified lines (See ICLAC register)	No commonly misidentified lines are used in this study.

Animals and other organisms

Policy information about studies involving animals; ARRIVE guidelines recommended for reporting animal research

Laboratory animals	C57BL/6J male mice were purchased from Vital River Animal Technology Co., Ltd. (Beijing, China) and Fto flox male mice (Stock No. 027830) were provided by the Jackson Laboratory. 12-16 week old mice were used for behavioral tests; 8-9 week old mice were used for establishing depression models; 12-16 week old mice were used for MeRIP-seq and gene-specific m6A qPCR.
Wild animals	No wild animals were involved in our study.
Field-collected samples	This study did not involve field-collected samples.
Ethics oversight	All animal work was performed in accordance with the institutional guidelines of the Beijing Administration Office of Laboratory and this study was approved by the Institutional Review Board of Chinese Academy of Medical Sciences and Peking Union Medical College

Note that full information on the approval of the study protocol must also be provided in the manuscript.

Human research participants

Policy information about studies involving human research participants

Population characteristics	We performed microarray analysis of the peripheral blood of 36 MDD patients (20 females and 16 males) aged 39.9 ± 12.5 (SD) years and 20 healthy controls (10 females and 10 males) aged 36.6 ± 11.1 (SD) years. We performed real-time quantitative PCR analysis of the peripheral blood of Fifty unrelated MDD patients (27 women and 23 men, aged 31.5 ± 9.8 (SD) years) and 50 healthy subjects (28 women and 22 men, aged 29.5 ± 8.3 (SD) years).<br>Hippocampal tissues were acquired from three patients with MDD and three healthy controls:<br>Subject 1 : Female, 87 years old<br>Subject 2 : Female, 86 years old<br>Subject 3 : Male, 72 years old<br>Healthy control subjects:<br>Subject1 : Female, 81 years old<br>Subject2 : Male, 81 years old<br>Subject 3 : Female, 87 years old
Recruitment	36 MDD patients and 20 healthy controls were recruited from the Department of Psychiatry, First Hospital of Shanxi Medical University, Taiyuan, China.. Fifty patients with MDD (27 women and 23 men) aged 31.5 ± 9.8 (SD) years and 50 healthy controls (28 women and 22 men) aged 29.5 ± 8.3 (SD) years were recruited to validate the result of microarray. Hippocampal tissues of the human brain were provided by the Human Brain Bank, Chinese Academy of Medical Sciences & Peking Union Medical College, Beijing, China. There was no self-selection bias in recruiting patients.
Ethics oversight	All subjects provided written informed consent prior to participating in this study, which was approved by the Ethics Committee of the Ethics Committee of the Chinese Academy of Medical Sciences and Peking Union Medical College.

Note that full information on the approval of the study protocol must also be provided in the manuscript.
